# Haematocrit During Cardiopulmonary Bypass and Risk for Acute Kidney Injury: An Observational Single-Centre Study

**DOI:** 10.1093/icvts/ivag207

**Published:** 2026-07-25

**Authors:** Anders Karl Hjärpe, Anders Jeppsson, Lukas Lannemyr, Birgitta Romlin

**Affiliations:** Department of Perfusion, Sahlgrenska University Hospital, Gothenburg SE-41345, Sweden; Department of Anaesthesia and Intensive Care, Institute of Clinical Sciences, Sahlgrenska Academy, University of Gothenburg, Gothenburg SE-41345, Sweden; Department of Cardiothoracic Surgery, Sahlgrenska University Hospital, Gothenburg SE-41345, Sweden; Department of Molecular and Clinical Medicine, Institute of Medicine, Sahlgrenska Academy, University of Gothenburg, Gothenburg SE-41345, Sweden; Department of Anaesthesia and Intensive Care, Institute of Clinical Sciences, Sahlgrenska Academy, University of Gothenburg, Gothenburg SE-41345, Sweden; Department of Cardiothoracic Anaesthesia, Sahlgrenska University Hospital, Gothenburg SE-41345, Sweden; Department of Anaesthesia and Intensive Care, Institute of Clinical Sciences, Sahlgrenska Academy, University of Gothenburg, Gothenburg SE-41345, Sweden; Department of Paediatric Anaesthesia and Intensive Care, Queen Silvia Children’s Hospital, Gothenburg SE-41650, Sweden

**Keywords:** acute kidney injury, cardiac surgery, cardiopulmonary bypass, haemoglobin, haematocrit

## Abstract

**Objectives:**

Low haematocrit during cardiopulmonary bypass is a known risk factor for acute kidney injury, but it is unclear whether high haematocrit also increases acute kidney injury risk. We examined if a haematocrit range associated with a reduced risk for acute kidney injury could be identified.

**Methods:**

This observational study included cardiac surgery patients operated on between 2016 and 2020, using prospectively collected data from the SWEDEHEART registry and a local cardiopulmonary bypass database. Acute kidney injury was defined by registry-available data as a postoperative serum‑creatinine increase >1.5 times baseline or postoperative initiation of renal replacement therapy. For each predefined haematocrit threshold, weighted exposure was calculated as the accumulated time‑and‑magnitude by which haematocrit rose above or fell below the thresholds. Associations between weighted exposure and acute kidney injury were analysed using multivariable logistic regression adjusted for established acute kidney injury risk factors.

**Results:**

A total of 3798 patients were included. Postoperative acute kidney injury occurred in 598 of 3798 patients (15.7%). After adjustment, a non‑linear association between haematocrit weighted exposure during aortic cross‑clamp and acute kidney injury was observed. AKI risk increased with haematocrit below 25% (adjusted odds ratio [aOR] 1.006 per 1 [%×min]; 95% CI 1.002-1.009; *P* < .001) and above 38% (aOR 1.006 per 1 [%×min]; 95% CI 1.001-1.011; *P* = .019).

**Conclusions:**

In this cohort, haematocrit exposure below 25% and above 38% during aortic cross-clamp was associated with higher postoperative acute kidney injury risk. Further studies are needed to confirm these findings.

## Graphical Abstract

**Figure:**
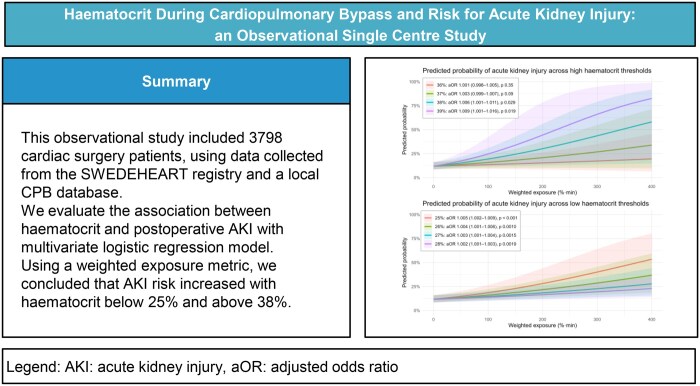

## INTRODUCTION

The techniques and equipment for cardiopulmonary bypass (CPB) used during cardiac surgery have evolved considerably in recent decades. The priming volume of the CPB circuit has been reduced, and because of this, the haemoglobin and haematocrit levels during CPB are higher today than before.^1^ This has led to a reduction in blood transfusion and complications related to low haemoglobin and haematocrit levels during and after cardiac surgery with CPB.^2^ However, some data suggest that high levels of haematocrit during and after CPB increase the risk of complications.^3^^,^^4^

One of the most common severe complications after cardiac surgery is acute kidney injury (AKI). The incidence varies between 5% and 30% depending on the surgical procedure and AKI definition used, and approximately 1% of cardiac surgery patients need postoperative renal replacement therapy.^5^^,^^6^ Even a mild increase in postoperative serum creatinine concentration has been linked to increased morbidity and mortality in cardiac surgery patients.^7^

Low preoperative haemoglobin concentration and haematocrit <24% during CPB are well-established risk factors for AKI after cardiac surgery.^8–10^ However, in a previous study, we observed a nonlinear relationship between preoperative haemoglobin concentration and AKI with an increased risk also in patients with high preoperative haemoglobin concentrations.^11^ It is not known if a haematocrit above the normal range during CPB is associated with an increased risk for AKI. Therefore, we investigated if a non-linear relationship between haematocrit during CPB and AKI risk exists.

## METHODS

### Ethical statement

The study was conducted according to the Declaration of Helsinki and was approved by the Regional Research Ethics Committee (approval no. 2020–06253). Given the registry-based study design, the committee waived individual patient consent.

### Study design and exclusion criteria

This was an observational single-centre study that utilized prospectively registered data from the Swedish Cardiac Surgery Registry,^12^ and the institutional CPB database at Sahlgrenska University Hospital, Gothenburg. To link individual patient data, the Swedish personal identification number was used. Exclusion criteria were preoperative dialysis, missing follow-up data, use of circulatory arrest, ventricular assist device and transplantation surgery, missing CPB data, and cases where cross-clamp was not used.

### Data sources

The Swedish Cardiac Surgery Registry is part of the SWEDEHEART registry.^12^ It contains detailed clinical and operative characteristic demographics and information along with short-term outcomes for all cardiac operations in Sweden since 1992. The CPB database contains data collected using the LivaNova Connect patient monitoring and data management system (LivaNova, London, United Kingdom). The system registers CPB data every 20 seconds automatically.

### Exposure and outcome definitions

#### Exposure variable

The primary exposure was haematocrit during cross-clamp time. Haematocrit was operationalized using threshold‑based weighted exposure metrics,^13^ capturing both the magnitude and the duration of deviations from predefined haematocrit level thresholds. Two continuous variables were constructed: low‑haematocrit weighted exposure (LHWE) and high‑haematocrit weighted exposure (HHWE). For each time interval during cross-clamp time, the deficit below the predefined low threshold or excess above the predefined high threshold was multiplied by the duration in minutes, resulting in a cumulative sum, expressed in %‑min (%·min).

As an example, if the low haematocrit threshold is 25% and the patient has a haematocrit of 20% for 10 minutes during cross‑clamp time, this results in a weighted exposure of 50 %·min. Likewise, if the patient has a haematocrit of 24% for 50 minutes, the weighted exposure is also 50 %·min.

#### Outcome variable

The outcome was postoperative AKI, defined by registry-available data as a serum creatinine increase >1.5 times from baseline value or initiation of renal replacement therapy. Baseline serum creatinine was defined as the last known value prior to surgery.

### CPB management

For all procedures, a Stöckert S5 heart-lung machine (LivaNova) and a CPB circuit consisting of an Inspire 8 or 8 F oxygenator (LivaNova) and an Inspire hard-shell venous reservoir (LivaNova) were used. The oxygenator and venous reservoir were coated with the PHISIO coating (LivaNova). The CPB circuit was primed with 1100 mL of Ringer’s acetate (Fresenius Kabi AB, Uppsala, Sweden) and 200 mL of 20% mannitol (Fresenius Kabi AB). An induction dose of 350-400 IU/kg of heparin was administered and supplemented to maintain activated clotting time >480 s. CPB was maintained with a roller pump and non-pulsatile flow at a target flow rate of 2.4-2.6 L/minute/m^2^. Target mean arterial pressure (MAP) was 50-70 mmHg. To regulate MAP, vasopressor therapy (norepinephrine/phenylephrine) and vasodilator therapy (nitroprusside) were used. Transfusion of packed red blood cells was administered if haematocrit was <24%. Cold-blood cardioplegia was used for cardioprotection. After decannulation, the heparin effect was reversed with protamine (0.8 mg/100 IU of heparin). Final decisions on CPB and anaesthesia management were always left to the anaesthetist, perfusionist, and surgeon in charge.

### Haematocrit measurement

Haematocrit was measured with the b-care or b-capta optical sensor (LivaNova, London, UK). The sensor was calibrated to blood samples collected from the CPB circuit and analysed on the RAPIDPoint 500e analyser (Siemens Healthineers, Erlangen, Germany) after cardioplegia administration.

### Statistical methods

Continuous data are presented as mean and standard deviation, median and interquartile range, or median and range, where appropriate. Categorical variables are reported as frequencies and percentages. For all analyses, a *P*-value of <0.05 was considered statistically significant. Data were analysed using R version 4.5.1 and RStudio version 2025.09.1 + 401.

To evaluate the association between haematocrit and postoperative AKI, we first constructed a multivariate logistic regression model with median haematocrit during cross-clamp time as a restrictive cubic spline adjusted for known risk factors for postoperative AKI.^8^ These were age, sex, body mass index (BMI), smoking status, dyspnoea (New York Heart Association [NYHA] class), preoperative diabetes, peripheral vascular disease, hypertension, estimated glomerular filtration rate (eGFR), critical preoperative event, left ventricular ejection fraction (LVEF), operative priority, and type of cardiac procedure. Variable definitions are presented in **Table S1**.

We then investigated whether inflection points for risk could be identified using the LHWE and HHWE metrics. For each candidate haematocrit threshold, we first constructed a multivariate logistic regression model including aortic cross-clamp time in addition to the original covariates. Based on these results, and guided by clinically relevant haematocrit levels during CPB, we performed a grid search across threshold pairs ranging from 25% to 27% for LHWE and 37% to 39% for HHWE. Each candidate model was evaluated using the area under the ROC curve, and calibration was evaluated using Hosmer-Lemeshow test. Models in which both LHWE and HHWE yielded adjusted odds ratios greater than 1.005 equal to a marginal effects estimation of 0.0005 that reached statistical significance were interpreted as a meaningful increase in risk. As an example, if the LHWE is 50 %·min, as in the earlier example, the increase in risk would be 2.5%. To evaluate the final model, a sensitivity analysis was performed in which we additionally included haematocrit reduction on CPB, MAP <40 mmHg during aortic cross-clamp time, MAP >90 mmHg during aortic cross-clamp time, SvO_2_ <65% during aortic cross-clamp, flow index during aortic cross-clamp <2 L/minute/m^2^, and mean flow index during aortic cross-clamp.

## RESULTS

### Study population

**Figure 1.**
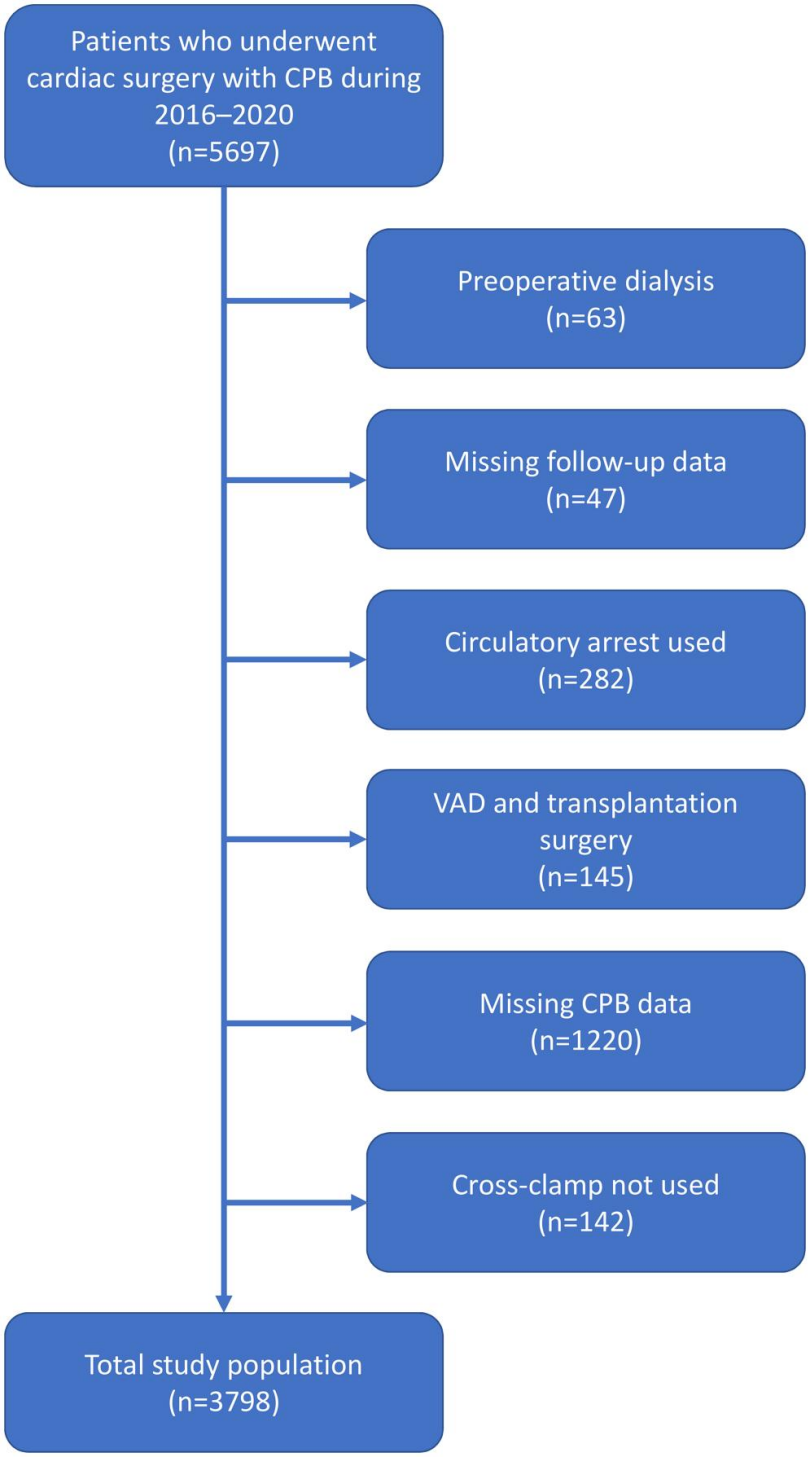
Flow Chart of Included and Excluded Patients. Abbreviation: CPB, cardiopulmonary bypass

A flow chart of included and excluded patients is depicted in **Figure 1**. A total of 5697 consecutive patients who underwent cardiac surgery with CPB during 2016-2020 were identified. After exclusion, the final study population consisted of 3798 patients. The patients who were missing follow-up data had no follow-up data available and were therefore excluded. The patients who had missing CPB data had no data registered in the CPB database and were also excluded. A comparison of patients with and without CPB data is presented in **Table S2**.

### General

Demographic data are presented in **Table 1** and **Table S3**. The mean age was 66 (12) years, and 24.4% of the patients were female. Of the 3798 patients, 1841 (48.6%) underwent isolated coronary artery bypass grafting (CABG), 1442 (38.1%) isolated valve, 215 (5.7%) combined CABG and valve, and 290 (7.7%) other or multiple procedures. The mean Logistic European System for Cardiac Operative Risk Evaluation (EuroSCORE) II was 1.71 (1.07, 3.16)% and the overall 30-day mortality was 2.1%. Of the 3798 patients, 598 (15.7%) developed postoperative AKI; of these, 93 (15.6%) needed postoperatively initiated dialysis. Baseline characteristics differed across haematocrit categories. Patients with haematocrit <25% were older, had lower preoperative haemoglobin and eGFR, and more often presented with higher NYHA class and urgent or emergency procedures. Those within the reference range showed the lowest 30‑day mortality. Patients with haematocrit >38% were younger, predominantly male, and had higher BMI, body surface area, and preoperative haemoglobin, and were more frequently elective cases. EuroSCORE II and mortality were highest in the <25% group.

**Table 1.** Demographic Data in Patients Stratified by Exposure to Haematocrit Levels

Variable	Overall *n* = 3798	<25% *n* = 1447	Within range *n* = 2023	>38% *n* = 328
Age (years), mean (SD)	66 (12)	69 (11)	66 (12)	62 (12)
Male sex (%)	2872 (75.6%)	827 (57.2%)	1735 (85.8%)	310 (94.5%)
BMI, mean (SD)	27.2 (4.5)	26.4 (4.4)	27.6 (4.4)	28.6 (4.8)
BSA, mean (SD)	1.98 (0.21)	1.88 (0.20)	2.02 (0.20)	2.11 (0.20)
Smoking status (%)				
Never smoked	1260 (39.9%)	475 (39.7%)	681 (40.3%)	104 (38.1%)
Former smoker	1542 (48.8%)	597 (49.9%)	817 (48.3%)	128 (46.9%)
Current smoker	358 (11.3%)	125 (10.4%)	192 (11.4%)	41 (15.0%)
Diabetes (%)	793 (20.9%)	319 (22.1%)	426 (21.1%)	48 (14.6%)
Peripheral vascular disease (%)	182 (4.8%)	82 (5.7%)	90 (4.5%)	10 (3.0%)
Hypertension (%)	2404 (65.8%)	924 (66.8%)	1281 (65.7%)	199 (62.8%)
Glomerular filtration rate, mean (SD)	68 (17)	64 (18)	70 (15)	72 (16)
Critical preoperative event (%)	82 (2.2%)	56 (3.9%)	17 (0.8%)	9 (2.7%)
Left ventricular ejection fraction (%)				
LVEF Good >50%	2783 (73.5%)	1063 (73.7%)	1490 (73.9%)	230 (70.8%)
LVEF Fair 30%-49%	802 (21.2%)	302 (20.9%)	428 (21.2%)	72 (22.2%)
LVEF Poor <30%	199 (5.3%)	77 (5.3%)	99 (4.9%)	23 (7.1%)
Operative priority (%)				
Elective	2176 (57.4%)	808 (56.0%)	1158 (57.3%)	210 (64.0%)
Urgent	1400 (36.9%)	520 (36.0%)	779 (38.5%)	101 (30.8%)
Emergency/salvage	217 (5.7%)	116 (8.0%)	84 (4.2%)	17 (5.2%)
Cardiac procedure (%)				
CABG only	1841 (48.6%)	615 (42.7%)	1105 (54.7%)	121 (36.9%)
Valve only	1442 (38.1%)	625 (43.4%)	664 (32.9%)	153 (46.6%)
CABG + valve	215 (5.7%)	95 (6.6%)	105 (5.2%)	15 (4.6%)
Other/multiple	290 (7.7%)	106 (7.4%)	145 (7.2%)	39 (11.9%)
Aortic cross‑clamp time, minutes, mean (SD)	66 (35)	69 (38)	62 (30)	74 (42)
Preoperative haemoglobin g/L, mean (SD)	137 (15)	129 (15)	141 (12)	150 (13)
30-day mortality (%)	79 (2.1)	49 (3.4)	23 (1.1)	7 (2.1)
Logistic EuroSCORE II (median [IQR])	1.71 [1.07, 3.16]	2.23 [1.31, 4.32]	1.50 [0.96, 2.57]	1.52 [0.93, 2.72]
Postoperative AKI	598 (15.7%)	306 (21.1%)	241 (11.9%)	51 (15.5%)

Abbreviations: BMI, body mass index; CABG, coronary artery bypass graft; eGFR, estimated glomerular filtration rate; EuroSCORE, European System for Cardiac Operative Risk Evaluation; LVEF, left ventricular ejection fraction; NYHA, New York Heart Association.

### Haematocrit and postoperative AKI

**Figure 2.**
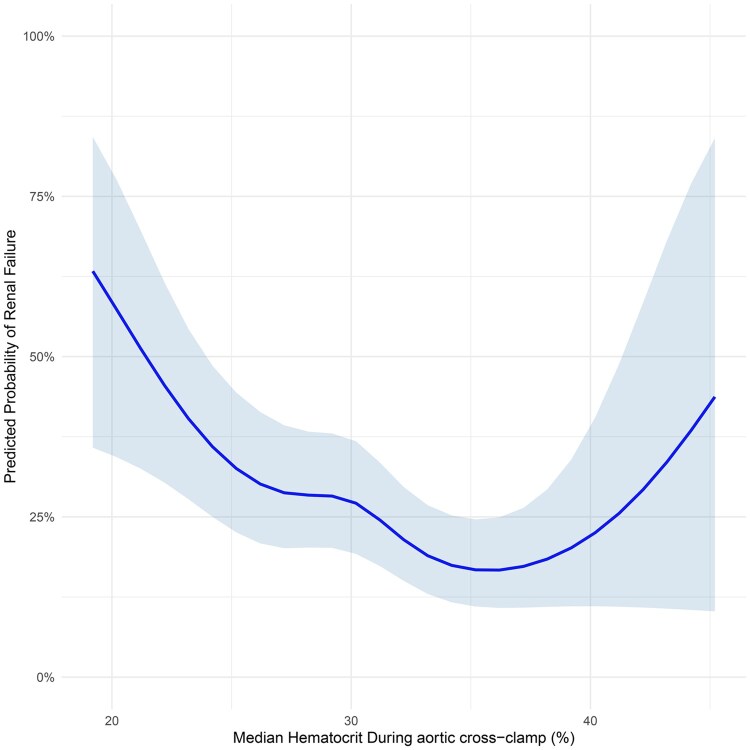
Predicted Probability of Acute Kidney Injury Across Median Haematocrit During Aortic Cross-Clamp Time. *Note*: Adjusted for age, sex, body mass index, smoking status, dyspnoea (New York Heart Association class), preoperative diabetes, peripheral vascular disease, hypertension, estimated glomerular filtration rate, critical preoperative event, left ventricular ejection fraction, operative priority, and type of cardiac procedure

The predicted probability of AKI for median haematocrit during aortic cross-clamp time is depicted in **Figure 2**. Median haematocrit during aortic cross-clamp time showed a U-shaped risk curve, with the lowest predicted probability between a median haematocrit of 34%-37%. A full multivariate model is presented in **Table S4**.

**Figure 3.**
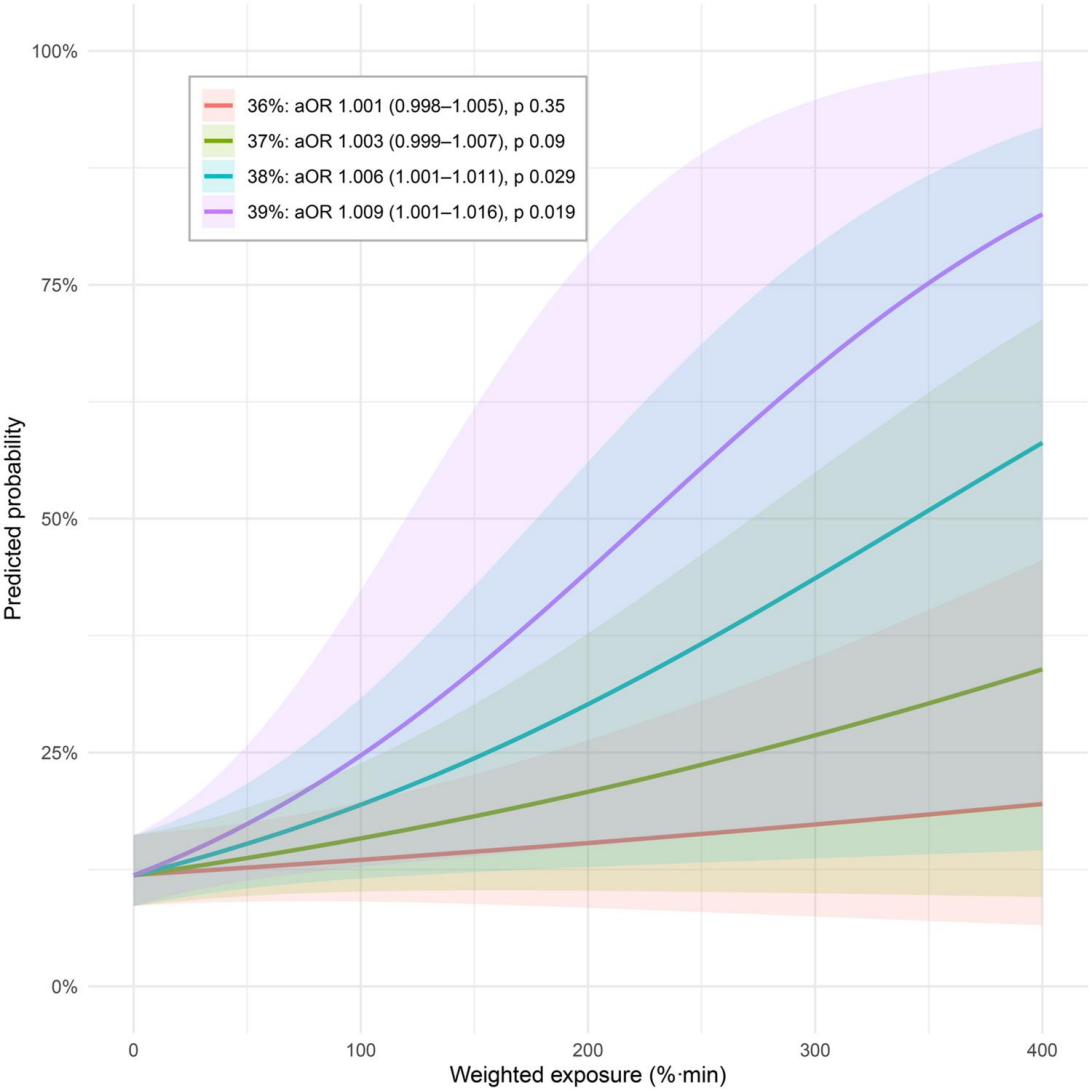
Predicted Probability of Acute Kidney Injury Across High Haematocrit Thresholds. *Note*: Adjusted for aortic cross-clamp time, age, sex, body mass index, smoking status, dyspnoea (New York Heart Association class), preoperative diabetes, peripheral vascular disease, hypertension, estimated glomerular filtration rate, critical preoperative event, left ventricular ejection fraction, operative priority, and type of cardiac procedure. Abbreviation: aOR, adjusted odds ratio

Distribution data for the haematocrit weighted exposure variables are presented in **Table S5**. The predicted probability of AKI across high haematocrit thresholds is depicted in **Figure 3**. The adjusted odds ratio (aOR) increased when raising the threshold, and at a haematocrit threshold of 38%, the predefined effect size was reached (aOR 1.006 per 1 [%×min]; 95% CI 1.001-1.011; *P* = .029).

**Figure 4.**
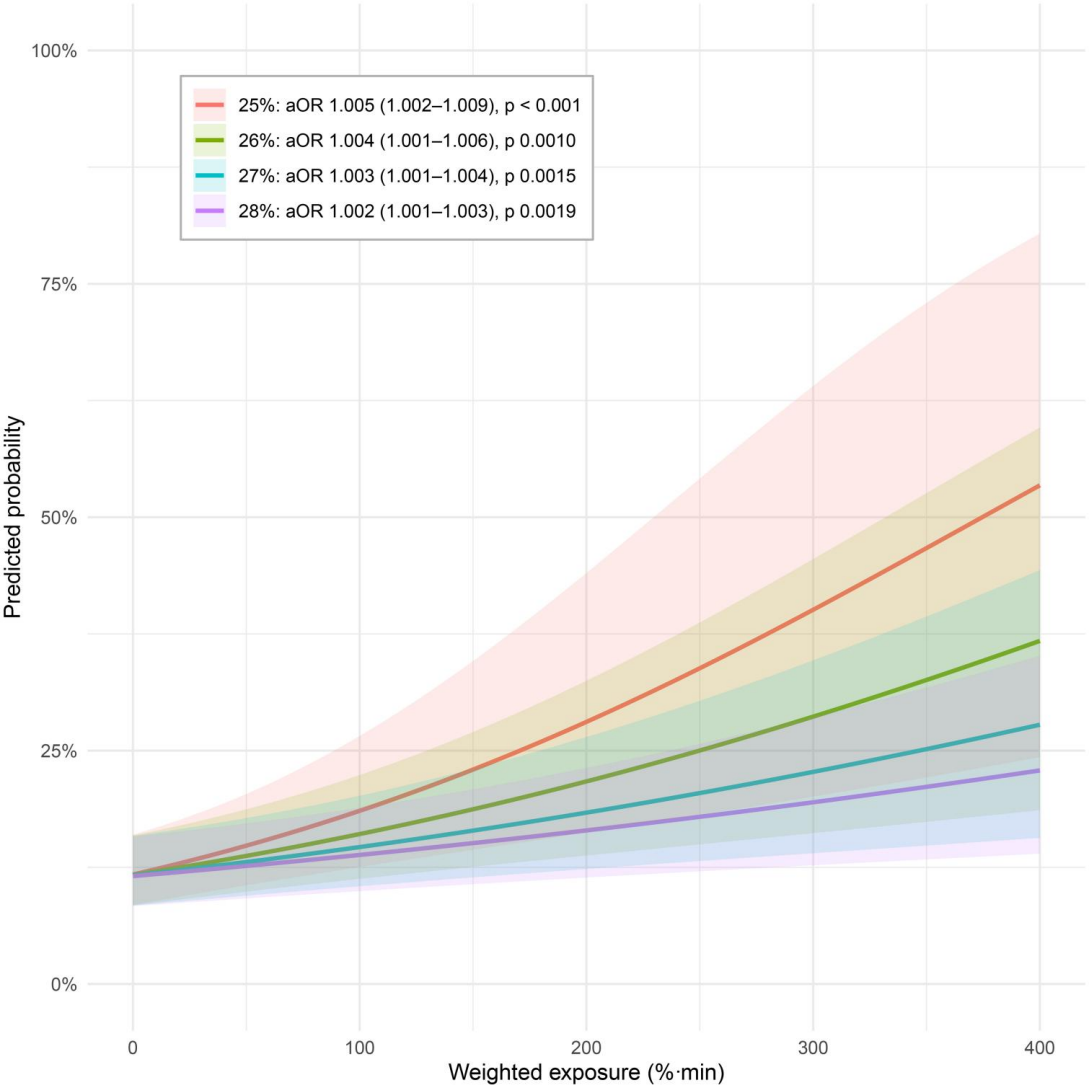
Predicted Probability of Acute Kidney Injury Across Low Haematocrit Thresholds. *Note*: Adjusted for aortic cross-clamp time, age, sex, body mass index, smoking status, dyspnoea (New York Heart Association class), preoperative diabetes, peripheral vascular disease, hypertension, estimated glomerular filtration rate, critical preoperative event, left ventricular ejection fraction, operative priority, and type of cardiac procedure. Abbreviation: aOR, adjusted odds ratio

Predicted probability of AKI across low haematocrit thresholds is depicted in **Figure 4**. The adjusted odds ratio increased when lowering the threshold, and at a haematocrit threshold of 25%, the predefined effect size was reached (aOR 1.005 per 1 [%×min]; 95% CI 1.002-1.009; *P* < .001).

After a grid search of candidate threshold values and evaluation of the area under the ROC curve and Hosmer-Lemeshow goodness-of-fit test (**Tables S6-S15**) the best combination remained 25% and 38%. The multivariate logistic regression model including both high- and low-threshold variables is presented in **Table 2** and **Table S11**. In the following sensitivity analysis (**Table S16**), effect sizes were approximately unchanged in both threshold values.

**Table 2.** Multivariable Logistic Regression for Associations Between Threshold Levels of Haematocrit During Cross-Clamp and Postoperative Acute Kidney Injury

Variable	Adjusted odds ratio (95% CI)	*P*-value	Marginal effects estimation (95% CI)	*P*-value
Low threshold weighted exposure HCT <25%	1.006 (1.002-1.009)	<.001	0.0006 (0.0003-0.0009)	<.001
High threshold weighted exposure HCT >38%	1.006 (1.001-1.011)	.019	0.0007 (0.0001-0.0012)	.019
AUC (95% CI)	0.7539 (0.7286-0.7792)			
Hosmer-Lemeshow test	χ^2^ = 5.49		*P*-value .70	

Adjusted for aortic cross-clamp time, age, sex, body mass index, smoking status, dyspnoea (New York Heart Association class), preoperative diabetes, peripheral vascular disease, hypertension, estimated glomerular filtration rate, critical preoperative event, left ventricular ejection fraction, operative priority, and type of cardiac procedure.

Abbreviations: AUC, Area Under the Curve; HCT, Haematocrit.

## DISCUSSION

The main findings of this study were that a haematocrit >38% during aortic cross‑clamp appears to be associated with a higher risk of postoperative AKI and that the use of a weighted exposure metric can provide additional analytical information by capturing both the magnitude and duration of haematocrit deviations.

### Acute kidney injury

Postoperative AKI is one of the most common and severe complications after cardiac surgery. In the present study, 598 (15.7%) of the patients developed postoperative AKI; of these, 93 (15.6%) needed postoperative-initiated dialysis. Postoperative AKI was associated with a greatly increased 30-day mortality (9.7% vs 0.7%), which is in line with previous studies.^11^^,^^14^

### Haematocrit and postoperative AKI

To investigate postoperative AKI and haematocrit levels during CPB, previous studies have focused on nadir haematocrit values or low threshold for oxygen delivery during CPB.^15–17^ Guidelines now recommend transfusion of red blood cells if haematocrit is below 18% and that it should be considered between 18% and 24%. It is also recommended that a goal-directed perfusion strategy aimed at limiting the nadir of DO_2_ and the length of CPB time with low DO_2_ values should be used.^18^ Oxygen delivery during CPB is calculated by multiplying the pump flow and oxygen content of arterial blood, and the oxygen content is highly dependent on the haematocrit value. Nadir haematocrit and low threshold for oxygen delivery can therefore give guidance for pump flow and the need for transfusion of red blood cells, but it gives no guidance for haemodilution. The definition of a high threshold has, to our knowledge, not previously been performed, but Ranucci et al have shown that a high nadir haematocrit during CPB increases the risk for AKI.^19^ To evaluate threshold values, we instead suggest that a weighted exposure metric should be used. Mukaida et al have previously used a weighted exposure method to evaluate oxygen delivery thresholds and found that this metric had higher accuracy predicting AKI than nadir value or time below threshold for oxygen delivery.^13^ To our knowledge, we are the first to investigate the haematocrit thresholds during CPB in this way.

In our study, we found that the median haematocrit during aortic cross-clamp time showed a U-shaped risk curve, but the median value does not tell us where a potential threshold value is located. When we investigated the threshold values with a weighted exposure metric, we found that haematocrit thresholds below 25% and above 38% were associated with a statistically significant association. The risk increases were similar in both high- and low-threshold levels and the low-threshold value of 25% is in line with established nadir values of 24%-26%.^9^^,^^16^^,^^18^

The pathophysiology behind the increased risk for AKI at higher haematocrit levels is not known but can be induced by increased shear stress in the CPB circuit with increased activation of blood components and increased haemolysis. This would in turn increase the micro-embolic load and the release of free haemoglobin which in turn would lead to impaired microcirculation and tissue oxygenation.^20^ On the other hand, a recent study has shown that the release of free haemoglobin is not associated with increased AKI.^21^

One can also hypothesize that the increased viscosity of the blood at higher haematocrit levels could intensify the strain on the heart in the immediate postoperative period which could in turn reduce cardiac output and oxygen delivery to the kidneys.^3^ Also, an increased viscosity can have a negative impact on the microcirculation, as it can create heterogeneous microcirculatory flow patterns, which can in turn lead to inadequate tissue oxygen delivery.^22^^,^^23^ During CPB, in which non‑pulsatile flow already challenges microcirculatory perfusion, deviations above the threshold value may shift the balance towards impaired microvascular oxygen delivery.

In this cohort, we observed an increased risk for AKI when the patient’s haematocrit rose above 38% during CPB. A high haematocrit not only affects AKI, but also increases the risk of oxygenator failure, as we have previously shown.^4^

### Limitations

The strengths of this study include the large, well-defined study population and the automated registration of variables during CPB every 20 seconds during surgery. This registration made calculation of haematocrit weighted exposure possible. The limitations of this study include the registry-based definition of AKI, as data on urine output and the time-point of postoperative serum creatinine analysis are not included. This reduces the applicability of the Kidney Disease Improving Global Outcomes (KDIGO) AKI definitions^24^ as we could not apply the criterion of urine output and stage 1 AKI definition of an absolute increase of ≥26.5 μmol/L within 48 hours. This may have reduced the sensitivity for detecting AKI. Furthermore, the inclusion of patients with low eGFR introduces a potential prevalence-incidence bias. Nevertheless, adjustment for baseline renal function in the multivariable model likely attenuates this concern. In the adjustment set from Birnie et al, 2 of the variables used were not present in our dataset. These were catheter-to-surgery and triple-vessel disease. Also, the effects of other intraoperative variables such as MAP, central venous pressure, SvO_2_, CPB flow index, oxygen delivery, haemodilution, and transfusion of blood components were not included in the primary analysis. Furthermore, 1220 patients lacked CPB data because the data management system was not activated. This systematic omission introduces potential selection bias. Due to these limitations and the lack of external validation, our findings on haematocrit thresholds should be interpreted with caution and considered cohort-specific and hypothesis-generating.

## CONCLUSIONS

Haematocrit remains a central component of perfusion management. In this study, a haematocrit range of 25%-38% during aortic cross‑clamp appears to be associated with a lower risk of postoperative AKI. The use of a weighted exposure metric can provide additional analytical information by capturing both the magnitude and duration of haematocrit deviations. Further studies are needed to validate these findings.

## Supplementary Material

ivag207_Supplementary_Data

## Data Availability

Data available on request.

## Abbreviations

AKI: acute kidney injury
BMI: body mass index
CABG: coronary artery bypass grafting
CPB: cardiopulmonary bypass
eGFR: estimated glomerular filtration rate
EuroSCORE: European System for Cardiac Operative Risk Evaluation
HHWE: high haematocrit weighted exposure
KDIGO: Kidney Disease Improving Global Outcomes
LHWE: low haematocrit weighted exposure
LVEF: left ventricular ejection fraction
MAP: mean arterial pressure
NYHA: New York Heart Association
SvO_2_: mixed venous oxygen saturation
