# Supplementary material for: Haematocrit During Cardiopulmonary Bypass and Risk for Acute Kidney Injury: An Observational Single-Centre Study
Source: Interdiscip Cardiovasc Thorac Surg. 2026 Jul 25;41(8):ivag207. doi: 10.1093/icvts/ivag207 (PMC13459969; doi:10.1093/icvts/ivag207)
Supplement: Supplementary file 1 [file ivag207_supplementary_data.docx]

**SUPPLEMENTARY MATERIALS**

**Supplementary Table 1, Variable definition.**

| **Variable** | **Variable definition** |
| --- | --- |
| Age | The patient age in years grouped into three categories <60 years, 60 to 74 years and ≥75 years. |
| Sex | The patient’s sex, male or female. |
| Body mass index, kg/m2 | The patient’s body mass index grouped into five categories: BMI <20.0, BMI 20.0 to 24.9, BMI 25.0 to 29.9, BMI 30.0 to 34.9 and BMI ≥35.0. |
| Smoking status | Patient smoking status grouped into three categories: never smoked, former smoker and current smoker. The patient was considered a former smoker if > 1 month had passed since quitting smoking. |
| NYHA class | Patient NYHA class: I = No limitation of physical activity. Ordinary activity does not cause undue fatigue, palpitation, or dyspnoea; II = Slight limitation of physical activity. Comfortable at rest, but ordinary activity results in fatigue, palpitation, or dyspnoea; III = Marked limitation of physical activity. Comfortable at rest, but less than ordinary activity causes fatigue, palpitation, or dyspnoea; IV = Unable to carry out any physical activity without discomfort. Symptoms of heart failure are present even at rest. |
| Diabetes | Patient has a known diabetes, regardless of the treatment given. |
| Peripheral vascular disease | The patient has one or more of the following: claudication, carotid occlusion or > 50% stenosis, previous or planned intervention on the abdominal aorta, collateral arteries or carotids. |
| Hypertension | The patient has a drug treatment for hypertension, now or in the past. |
| Preoperative haemoglobin | Patients latest haemoglobin value taken before surgery. grouped into three categories <100 g/L, 100 to 119 g/L and ≥120 g/L |
| Glomerular filtration rate | Patients estimated creatinine clearance calculated with the LRM18 formula. grouped into four categories eGFR <30.0, eGFR 30.0 to 59.9, eGFR 60.0 to 89.9, eGFR ≥ 90. |
| Critical preoperative event | The patient preoperatively experienced one or more of the following: ventricular tachycardia or fibrillation or cardiac resuscitation, preoperative cardiac massage, preoperative ventilation before arrival at the operating room, preoperative inotropic support, aortic balloon user or preoperative acute renal failure (anuria or oliguria < 10ml/hr). |
| Left ventricular ejection fraction | Left ventricular ejection fraction grouped into three categories: good (50+ %), fair (30-49%), poor (<30%). |
| Operative priority | Operative priority grouped into three categories: Elective, planned procedure according to normal procedures; Urgent, patients who were not admitted for elective surgery but require surgery during the stay for medical reasons or patients who cannot be sent home without definitive action; Emergency/salvage: surgery before the next working day after the decision to operate or patients who require ongoing CPR on the way into the operating room or during anaesthesia (Does not include patients who require CPR after induction of anaesthesia). |
| Cardiac procedures | Type of procedure grouped into four categories: CABG only, Valve only, CABG + valve, Other/multiple. |
|  |  |
| Aortic cross‑clamp time | Total aortic cross‑clamp time in minutes during surgery (if several the sum of multiple cross‑clamp times). |
| Haematocrit reduction on cardiopulmonary bypass | Calculated as the difference between preoperative haematocrit and mean haematocrit during aortic cross-clamp time. Theoretically this value reflects the total hemodilution seen during cardiopulmonary bypass. |
| Mean arterial pressure <40 mmHg during aortic cross‑clamp time, min | The time in minutes that the patient experienced a mean arterial pressure below 40 mmHg. |
| Mean arterial pressure >90 mmHg during aortic cross‑clamp time, min | The time in minutes that the patient experienced a mean arterial pressure above 90 mmHg. |
| SvO2 <65% during CPB, min | The time in minutes that the patient experienced a mixed venous oxygen saturation below 65%. |
| Flow index during aortic cross-clamp < 2L/min/m2, min | The time in minutes that the patient experienced a flow index < 2L/min/m2 during aortic cross-clamp time. |
| Mean Flow index during CPB, L/min/m^2^ | The mean flow index during CPB in L/min/m2. |

**Supplementary table 2, Demographic data in patients with and without CPB data**

| **Variable** | **Overall** n=5160 | **No CPB data** n=1220 | **CPB data** n=3940 | ***p* value** |
| --- | --- | --- | --- | --- |
| Age, years (mean (SD)) | 66 (12) | 66 (12) | 66 (12) | 0.742 |
| Age, factor (%) |  |  |  |  |
| Age <65 years | 1216 (23.6) | 295 (24.2) | 921 (23.4) | 0.833 |
| Age 65–74 years | 2619 (50.8) | 612 (50.2) | 2007 (50.9) |  |
| Age ≥75 years | 1325 (25.7) | 313 (25.7) | 1012 (25.7) |  |
| Male sex (%) | 3908 (75.7) | 945 (77.5) | 2963 (75.2) |  |
|  |  |  |  |  |
| BMI (mean (SD)) | 27.2 (4.5) | 27.3 (4.6) | 27.2 (4.5) | 0.909 |
| BMI, factor (%) |  |  |  |  |
| BMI <20 kg/m² | 162 (3.1) | 37 (3.0) | 125 (3.2) | 0.526 |
| BMI 20–24.9 kg/m² | 1497 (29.0) | 372 (30.5) | 1125 (28.6) |  |
| BMI 25–29.9 kg/m² | 2259 (43.8) | 516 (42.4) | 1743 (44.2) |  |
| BMI 30–34.9 kg/m² | 942 (18.3) | 216 (17.7) | 726 (18.4) |  |
| BMI ≥35 kg/m² | 297 (5.8) | 77 (6.3) | 220 (5.6) |  |
| BSA (mean (SD)) | 1.97 (0.21) | 1.96 (0.22) | 1.97 (0.21) | 0.641 |
| Smoking status (%) |  |  |  |  |
| Never smoked | 1710 (40.0) | 393 (39.4) | 1317 (40.2) | 0.464 |
| Former smoker | 2067 (48.4) | 478 (47.9) | 1589 (48.5) |  |
| Current smoker | 497 (11.6) | 127 (12.7) | 370 (11.3) |  |
| Diabetes (%) | 1082 (21.0) | 261 (21.4) | 821 (20.9) | 0.715 |
| Peripheral vascular disease (%) | 244 (4.7) | 51 (4.2) | 193 (4.9) | 0.335 |
| Hypertension (%) | 3268 (65.6) | 791 (66.2) | 2477 (65.4) |  |
| Serum creatinine (mean (SD)) | 88.97 (34.11) | 85.97 (33.07) | 89.90 (34.38) | <0.001 |
| Glomerular filtration rate (mean (SD)) | 68.5 (16.8) | 70.6 (16.4) | 67.9 (16.9) | <0.001 |
| Glomerular filtration rate, factor (%) |  |  |  |  |
| eGFR <30 mL/min/1.73 m^2^ | 130 (2.5) | 25 (2.1) | 105 (2.7) | <0.001 |
| eGFR 30–59.9 mL/min/1.73 m^2^ | 1262 (24.5) | 252 (20.7) | 1010 (25.7) |  |
| eGFR 60–89.9 mL/min/1.73 m^2^ | 3339 (64.8) | 815 (67.0) | 2524 (64.2) |  |
| eGFR ≥90 mL/min/1.73 m^2^ | 419 (8.1) | 125 (10.3) | 294 (7.5) |  |
| Critical preoperative event (%) | 122 (2.4) | 37 (3.3) | 85 (2.2) |  |
| Left ventricular ejection fraction (%) |  |  |  |  |
| LVEF Good >50% | 3681 (73.2) | 795 (72.1) | 2886 (73.5) | 0.575 |
| LVEF Fair 30-49% | 1079 (21.5) | 243 (22.1) | 836 (21.3) |  |
| LVEF Poor <30% | 267 (5.3) | 64 (5.8) | 203 (5.2) |  |
| Operative priority (%) |  |  |  |  |
| Elective | 2933 (56.9) | 671 (55.0) | 2262 (57.5) | 0.001 |
| Urgent | 1886 (36.6) | 442 (36.3) | 1444 (36.7) |  |
| Emergency/salvage | 334 (6.5) | 106 (8.7) | 228 (5.8) |  |
| Cardiac procedure (%) |  |  |  |  |
| CABG only | 2421 (47.0) | 530 (43.6) | 1891 (48.1) | 0.001 |
| Valve only | 1962 (38.1) | 479 (39.4) | 1483 (37.7) |  |
| CABG + valve | 323 (6.3) | 102 (8.4) | 221 (5.6) |  |
| Other/multiple | 440 (8.6) | 105 (8.6) | 335 (8.5) |  |
| Aortic cross‑clamp time (mean (SD)) | 66.43 (34.89) | 70.24 (34.36) | 65.25 (34.97) | <0.001 |
| Preoperative haemoglobin (mean (SD)) | 137.86 (15.32) | 139.02 (15.90) | 137.50 (15.12) | 0.002 |
| Preoperative haemoglobin, factor (%) |  |  |  |  |
| Hb >120 g/L | 4532 (88.1) | 1073 (88.2) | 3459 (88.0) | 0.713 |
| Hb 100–119 g/L | 551 (10.7) | 126 (10.4) | 425 (10.8) |  |
| Hb <100 g/L | 62 (1.2) | 17 (1.4) | 45 (1.1) |  |
| 30-day mortality (%) | 143 (2.8) | 61 (5.1) | 82 (2.1) | <0.001 |
| Logistic Euroscore II (median [IQR]) | 1.73 [1.08, 3.16] | 1.74 [1.10, 2.99] | 1.73 [1.08, 3.19] | 0.693 |
| Abbreviations: BMI, body mass index; CABG, coronary artery bypass graft; eGFR, estimated glomerular filtration rate; Hb, haemoglobin; HCT, haematocrit; LVEF, left ventricular ejection fraction; NYHA, New York Heart Association.  Note: Statistical tests for group differences; categorical variables chi-square test, Normally distributed continuous variables Student´s t-test, nonnormally distributed continuous variables Mann-Whitney’s U-test. | | | | |

**Table 3.
Demographic data in patients stratified by exposure to haematocrit levels**

| **Variable** | **Overall** n=3798 | **<25%** n=1447 | **Within range** n=2023 | **>38%** n=328 |
| --- | --- | --- | --- | --- |
| Age, years (mean (SD)) | 66 (12) | 69 (11) | 66 (12) | 62 (12) |
| Age, factor (%) |  |  |  |  |
| Age <65 years | 863 (22.7%) | 239 (16.5%) | 501 (24.8%) | 123 (37.5%) |
| Age 65–74 years | 1949 (51.3%) | 726 (50.2%) | 1066 (52.7%) | 157 (47.9%) |
| Age ≥75 years | 986 (26.0%) | 482 (33.3%) | 456 (22.5%) | 48 (14.6%) |
| Male sex (%) | 2872 (75.6%) | 827 (57.2%) | 1735 (85.8%) | 310 (94.5%) |
| BMI (mean (SD)) | 27.2 (4.5) | 26.4 (4.4) | 27.6 (4.4) | 28.6 (4.8) |
| BMI, factor (%) |  |  |  |  |
| BMI <20 kg/m² | 115 (3.0%) | 70 (4.8%) | 43 (2.1%) | 2 (0.6%) |
| BMI 20–24.9 kg/m² | 1089 (28.7%) | 505 (34.9%) | 517 (25.6%) | 67 (20.4%) |
| BMI 25–29.9 kg/m² | 1684 (44.4%) | 601 (41.6%) | 928 (45.9%) | 155 (47.3%) |
| BMI 30–34.9 kg/m² | 700 (18.4%) | 209 (14.5%) | 422 (20.9%) | 69 (21.0%) |
| BMI ≥35 kg/m² | 209 (5.5%) | 61 (4.2%) | 113 (5.6%) | 35 (10.7%) |
| BSA (mean (SD)) | 1.98 (0.21) | 1.88 (0.20) | 2.02 (0.20) | 2.11 (0.20) |
| Smoking status (%) |  |  |  |  |
| Never smoked | 1260 (39.9%) | 475 (39.7%) | 681 (40.3%) | 104 (38.1%) |
| Former smoker | 1542 (48.8%) | 597 (49.9%) | 817 (48.3%) | 128 (46.9%) |
| Current smoker | 358 (11.3%) | 125 (10.4%) | 192 (11.4%) | 41 (15.0%) |
| Diabetes (%) | 793 (20.9%) | 319 (22.1%) | 426 (21.1%) | 48 (14.6%) |
| Peripheral vascular disease (%) | 182 (4.8%) | 82 (5.7%) | 90 (4.5%) | 10 (3.0%) |
| Hypertension (%) | 2404 (65.8%) | 924 (66.8%) | 1281 (65.7%) | 199 (62.8%) |
| Serum creatinine (mean (SD)) | 90 (35) | 93 (45) | 88 (27) | 89 (26) |
| Glomerular filtration rate (mean (SD)) | 68 (17) | 64 (18) | 70 (15) | 72 (16) |
| Glomerular filtration rate, factor (%) |  |  |  |  |
| eGFR <30 mL/min/1.73 m^2^ | 103 (2.7%) | 78 (5.4%) | 20 (1.0%) | 5 (1.5%) |
| eGFR 30–59.9 mL/min/1.73 m^2^ | 978 (25.8%) | 470 (32.6%) | 444 (22.0%) | 64 (19.5%) |
| eGFR 60–89.9 mL/min/1.73 m2 | 2433 (64.2%) | 820 (56.8%) | 1400 (69.3%) | 213 (64.9%) |
| eGFR ≥90 mL/min/1.73 m2 | 277 (7.3%) | 75 (5.2%) | 156 (7.7%) | 46 (14.0%) |
| Critical preoperative event (%) | 82 (2.2%) | 56 (3.9%) | 17 (0.8%) | 9 (2.7%) |
| Left ventricular ejection fraction (%) |  |  |  |  |
| LVEF Good >50% | 2783 (73.5%) | 1063 (73.7%) | 1490 (73.9%) | 230 (70.8%) |
| LVEF Fair 30-49% | 802 (21.2%) | 302 (20.9%) | 428 (21.2%) | 72 (22.2%) |
| LVEF Poor <30% | 199 (5.3%) | 77 (5.3%) | 99 (4.9%) | 23 (7.1%) |
| Operative priority (%) |  |  |  |  |
| Elective | 2176 (57.4%) | 808 (56.0%) | 1158 (57.3%) | 210 (64.0%) |
| Urgent | 1400 (36.9%) | 520 (36.0%) | 779 (38.5%) | 101 (30.8%) |
| Emergency/salvage | 217 (5.7%) | 116 (8.0%) | 84 (4.2%) | 17 (5.2%) |
| Cardiac procedure (%) |  |  |  |  |
| CABG only | 1841 (48.6%) | 615 (42.7%) | 1105 (54.7%) | 121 (36.9%) |
| Valve only | 1442 (38.1%) | 625 (43.4%) | 664 (32.9%) | 153 (46.6%) |
| CABG + valve | 215 (5.7%) | 95 (6.6%) | 105 (5.2%) | 15 (4.6%) |
| Other/multiple | 290 (7.7%) | 106 (7.4%) | 145 (7.2%) | 39 (11.9%) |
| Aortic cross‑clamp time, min (mean (SD)) | 66 (35) | 69 (38) | 62 (30) | 74 (42) |
| Preoperative haemoglobin g/L (mean (SD)) | 137 (15) | 129 (15) | 141 (12) | 150 (13) |
| Preoperative haemoglobin, factor (%) |  |  |  |  |
| Hb >120 g/L | 3343 (88.3%) | 1088 (75.4%) | 1931 (95.7%) | 324 (99.1%) |
| Hb 100–119 g/L | 400 (10.6%) | 315 (21.8%) | 82 (4.1%) | 3 (0.9%) |
| Hb <100 g/L | 45 (1.2%) | 40 (2.8%) | 5 (0.2%) | 0 (0.0%) |
| Estimated haemodilution (mean (SD)) | -10.3 (3.4) | -10.7 (3.8) | -10.2 (3.1) | -8.8 (3.2) |
| MAP <40 mmHg during Aortic cross‑clamp, min (median [range]) | 1.00 [0.00, 150.33] | 1.67 [0.00, 150.33] | 0.67 [0.00, 101.33] | 0.67 [0.00, 75.00] |
| MAP >90 mmHg during Aortic cross‑clamp, min (median [range]) | 0 [0, 66] | 0 [0, 66] | 0 [0, 62] | 0 [0, 45] |
| SvO_2_ <65% during Aortic cross‑clamp, min (median [range]) | 0 [0, 82] | 0. [0, 82] | 0 [0, 81] | 0 [0, 13] |
| Flow index during aortic cross-clamp  < 2L/min/m2, min (median [range]) | 0 [0, 92] | 0 [0, 76] | 0 [0, 70] | 0 [0, 92] |
| Median Flow index during CPB, L/min/m2 (mean (SD)) | 2.55 (0.15) | 2.57 (0.17) | 2.54 (0.13) | 2.54 (0.15) |
| 30-day mortality (%) | 79 (2.1) | 49 (3.4) | 23 (1.1) | 7 (2.1) |
| Logistic EuroSCORE II (median [IQR]) | 1.71 [1.07, 3.16] | 2.23 [1.31, 4.32] | 1.50 [0.96, 2.57] | 1.52 [0.93, 2.72] |
|  |  |  |  |  |
| Postoperative AKI | 598 (15.7%) | 306 (21.1%) | 241 (11.9%) | 51 (15.5%) |
| Postoperative AKI level (%) |  |  |  |  |
| Level 1 | 321 (8.5%) | 147 (10.2%) | 146 (7.2%) | 28 (8.5%) |
| Level 2 | 152 (4.0%) | 86 (5.9%) | 55 (2.7%) | 11 (3.4%) |
| Level 3 | 125 (3.3%) | 73 (5.0%) | 40 (2.0%) | 12 (3.7%) |
| Abbreviations: AKI, acute kidney injury; BMI, body mass index; CABG, coronary artery bypass graft; eGFR, estimated glomerular filtration rate; Hb, haemoglobin; HCT, haematocrit; LVEF, left ventricular ejection fraction; MAP, mean arterial pressure NYHA, New York Heart Association; SvO2, mixed venous oxygen saturation. | | | | |

**Supplementary table 4.
Multivariate** **logistic regression model for associations between median haematocrit during aortic cross-clamp time** **and postoperative acute kidney injury.**

| **Variable** | **Adjusted odds ratio (95% CI)** | ***p*-value** |
| --- | --- | --- |
| Median Haematocrit during Aortic Cross-Clamp |  |  |
| Restrictive cubic spline 1 | 0.245 (0.092-0.678) per 1 % increase | 0.0057 |
| Restrictive cubic spline 2 | 0.118 (0.047-0.299) per 1 % increase | < 0.001 |
| Restrictive cubic spline 3 | 0.046 (0.00411-0.535) per 1 % increase | 0.013 |
| Restrictive cubic spline 4 | 1.076 (0.12-7.77) per 1 % increase | 0.94 |
| Aortic cross‑clamp time | 1.013 (1.01-1.017) per 1 min increase | < 0.001 |
| Age |  |  |
| Age <65 years | Reference |  |
| Age 65–74 years | 2.87 (1.936-4.34) | < 0.001 |
| Age ≥75 years | 4.57 (2.94-7.22) | < 0.001 |
| Male sex | 1.032 (0.777-1.376) | 0.83 |
| BMI |  |  |
| BMI <20 kg/m² | 0.794 (0.373-1.551) | 0.52 |
| BMI 20–24.9 kg/m² | 0.719 (0.54-0.953) | 0.023 |
| BMI 25–29.9 kg/m² | Reference |  |
| BMI 30–34.9 kg/m² | 1.323 (0.984-1.77) | 0.06 |
| BMI ≥35 kg/m² | 2.01 (1.264-3.15) | 0.0026 |
| Smoking status |  |  |
| Never smoked | Reference |  |
| Former smoker | 0.927 (0.729-1.18) | 0.54 |
| Current smoker | 0.637 (0.41-0.967) | 0.039 |
| NYHA class |  |  |
| NYHA I | Reference |  |
| NYHA II | 0.683 (0.498-0.938) | 0.018 |
| NYHA III | 1.186 (0.839-1.681) | 0.34 |
| NYHA IV | 1.747 (0.985-3.05) | 0.05 |
| Diabetes | 1.144 (0.871-1.494) | 0.33 |
| Peripheral vascular disease | 1.435 (0.903-2.23) | 0.12 |
| Hypertension | 1.449 (1.103-1.917) | 0.0084 |
| Preoperative haemoglobin |  |  |
| Hb >120 g/L | Reference |  |
| Hb 100-119 g/L | 1.272 (0.882-1.82) | 0.19 |
| Hb <100 g/L | 2.45 (0.943-6.27) | 0.06 |
| Glomerular filtration rate |  |  |
| eGFR <30 mL/min/1.73 m^2^ | 2.048 (1.146-3.61) | 0.014 |
| eGFR 30–59.9 mL/min/1.73 m^2^ | 0.628 (0.476-0.825) | < 0.001 |
| eGFR 60–89.9 mL/min/1.73 m^2^ | Reference |  |
| eGFR ≥90 mL/min/1.73 m^2^ | 2.88 (1.811-4.53) | < 0.001 |
| Critical preoperative event | 2.32 (0.886-6.12) | 0.09 |
| Left ventricular ejection fraction |  |  |
| LVEF Good >50% | Reference |  |
| LVEF Fair 30-49% | 1.89 (1.46-2.44) | < 0.001 |
| LVEF Poor <30% | 2.49 (1.563-3.91) | < 0.001 |
| Operative priority |  |  |
| Elective | Reference |  |
| Urgent | 0.874 (0.663-1.151) | 0.34 |
| Emergency/salvage | 1.371 (0.723-2.51) | 0.32 |
| Cardiac procedure |  |  |
| CABG only | Reference |  |
| Valve only | 0.948 (0.686-1.31) | 0.75 |
| CABG + valve | 0.916 (0.564-1.466) | 0.72 |
| Other/multiple | 1.314 (0.764-2.21) | 0.31 |
| Abbreviations: BMI, body mass index; CABG, coronary artery bypass graft; eGFR, estimated glomerular filtration rate; Hb, haemoglobin; HCT, haematocrit; LVEF, left ventricular ejection fraction; MAP, mean arterial pressure NYHA, New York Heart Association. | | |

**Supplementary table 5, Distribution of haematocrit threshold weighted exposure values**

| **Variable** | **Median[range]** | **>1 %·min** | **>100 %·min** | **>200 %·min** | **>300 %·min** | **>400 %·min** |
| --- | --- | --- | --- | --- | --- | --- |
| Low threshold weighted exposure HCT <30% | 47 [0, 1375] | 3,052 | 1,309 | 690 | 342 | 153 |
| Low threshold weighted exposure HCT <29% | 26 [0, 1134] | 2,775 | 1,001 | 461 | 184 | 89 |
| Low threshold weighted exposure HCT <28% | 10 [0, 961] | 2,481 | 716 | 273 | 98 | 49 |
| Low threshold weighted exposure HCT <27% | 3 [0, 800] | 2,149 | 461 | 134 | 47 | 20 |
| Low threshold weighted exposure HCT <26% | 1 [0, 645] | 1,778 | 246 | 57 | 25 | 9 |
| Low threshold weighted exposure HCT <25% | 0 [0, 491] | 1,447 | 114 | 28 | 7 | 2 |
| High threshold weighted exposure HCT >36% | 0 [0, 613] | 825 | 98 | 25 | 8 | 3 |
| High threshold weighted exposure HCT >37% | 0 [0, 474] | 573 | 45 | 9 | 3 | 1 |
| High threshold weighted exposure HCT >38% | 0 [0, 337] | 394 | 25 | 6 | 1 | 0 |
| High threshold weighted exposure HCT >39% | 0 [0, 215] | 264 | 11 | 3 | 0 | 0 |
| High threshold weighted exposure HCT >40% | 0 [0, 176] | 191 | 5 | 0 | 0 | 0 |

**Supplementary table 6, Multivariate model Haematocrit threshold weighted exposure <27% >37%**

| **Variable** | **Adjusted odds ratio (95% CI)** | **p-value** | **Marginal effects estimation (95% CI)** | **p-value** |
| --- | --- | --- | --- | --- |
| Low threshold weighted exposure HCT <27% | 1.003 (1.001-1.004) | < 0.001 | 0.0003 (0.0001-0.0005) | < 0.001 |
| High threshold weighted exposure HCT >37% | 1.004 (1-1.008) | 0.05 | 0.0004 (-0.0008) | 0.05 |
| Aortic cross‑clamp time | 1.012 (1.008-1.015) | < 0.001 | 0.0013 (0.0009-0.0016) | < 0.001 |
| Preoperative haemoglobin |  |  |  |  |
| Hb >120 g/L | Reference |  |  |  |
| Hb 100-119 g/L | 1.385 (0.964-1.97) | 0.07 | 0.038 (-0.0068-0.084) | 0.10 |
| Hb <100 g/L | 2.67 (1.004-6.96) | 0.045 | 0.137 (-0.027-0.301) | 0.10 |
| Age |  |  |  |  |
| Age <65 years | Reference |  |  |  |
| Age 65–74 years | 3.03 (2.04-4.57) | < 0.001 | 0.09 (0.063-0.117) | < 0.001 |
| Age ≥75 years | 5.23 (3.38-8.25) | < 0.001 | 0.161 (0.122-0.2) | < 0.001 |
| Male sex | 0.875 (0.667-1.152) | 0.34 | -0.015 (-0.046-0.016) | 0.35 |
| BMI |  |  |  |  |
| BMI <20 kg/m² | 0.813 (0.381-1.592) | 0.57 | -0.021 (-0.09-0.047) | 0.55 |
| BMI 20–24.9 kg/m² | 0.77 (0.579-1.018) | 0.07 | -0.026 (-0.054-0.0014) | 0.06 |
| BMI 25–29.9 kg/m² | Reference |  |  |  |
| BMI 30–34.9 kg/m² | 1.335 (0.997-1.782) | 0.05 | 0.034 (-0.0011-0.069) | 0.06 |
| BMI ≥35 kg/m² | 1.966 (1.236-3.07) | 0.0035 | 0.088 (0.021-0.155) | 0.0098 |
| Smoking status |  |  |  |  |
| Never smoked | Reference |  |  |  |
| Former smoker | 0.932 (0.734-1.184) | 0.56 | -0.0079 (-0.035-0.019) | 0.56 |
| Current smoker | 0.645 (0.418-0.976) | 0.043 | -0.044 (-0.084--0.0047) | 0.028 |
| NYHA class |  |  |  |  |
| NYHA I | Reference |  |  |  |
| NYHA II | 0.658 (0.481-0.902) | 0.0089 | -0.044 (-0.078--0.0095) | 0.012 |
| NYHA III | 1.161 (0.822-1.641) | 0.40 | 0.018 (-0.024-0.061) | 0.40 |
| NYHA IV | 1.676 (0.949-2.91) | 0.07 | 0.07 (-0.012-0.152) | 0.09 |
| Diabetes | 1.167 (0.891-1.52) | 0.26 | 0.017 (-0.013-0.048) | 0.27 |
| Peripheral vascular disease | 1.483 (0.937-2.3) | 0.08 | 0.048 (-0.012-0.107) | 0.12 |
| Hypertension | 1.439 (1.097-1.899) | 0.0092 | 0.038 (0.011-0.066) | 0.0065 |
| Glomerular filtration rate |  |  |  |  |
| eGFR <30 mL/min/1.73 m2 | 2.23 (1.256-3.92) | 0.0055 | 0.111 (0.019-0.202) | 0.018 |
| eGFR 30–59.9 mL/min/1.73 m2 | 0.646 (0.489-0.847) | 0.0018 | -0.043 (-0.069--0.018) | < 0.001 |
| eGFR 60–89.9 mL/min/1.73 m^2^ | Reference |  |  |  |
| eGFR ≥90 mL/min/1.73 m2 | 2.85 (1.8-4.46) | < 0.001 | 0.152 (0.075-0.229) | < 0.001 |
| Critical preoperative event | 2.34 (0.881-6.22) | 0.09 | 0.116 (-0.044-0.276) | 0.15 |
| Left ventricular ejection fraction |  |  |  |  |
| LVEF Good >50% | Reference |  |  |  |
| LVEF Fair 30-49% | 1.879 (1.454-2.42) | < 0.001 | 0.075 (0.042-0.107) | < 0.001 |
| LVEF Poor <30% | 2.45 (1.542-3.85) | < 0.001 | 0.114 (0.045-0.184) | 0.0013 |
| Operative priority |  |  |  |  |
| Elective | Reference |  |  |  |
| Urgent | 0.877 (0.665-1.154) | 0.35 | -0.014 (-0.043-0.015) | 0.34 |
| Emergency/salvage | 1.376 (0.728-2.51) | 0.31 | 0.039 (-0.042-0.12) | 0.35 |
| Cardiac procedure |  |  |  |  |
| CABG only | Reference |  |  |  |
| Valve only | 0.919 (0.666-1.269) | 0.61 | -0.0091 (-0.044-0.026) | 0.61 |
| CABG + valve | 0.883 (0.545-1.409) | 0.61 | -0.013 (-0.063-0.036) | 0.60 |
| Other/multiple | 1.254 (0.734-2.09) | 0.40 | 0.027 (-0.038-0.091) | 0.42 |
| Abbreviations: BMI, body mass index; CABG, coronary artery bypass graft; eGFR, estimated glomerular filtration rate; Hb, haemoglobin; HCT, haematocrit; LVEF, left ventricular ejection fraction; MAP, mean arterial pressure NYHA, New York Heart Association. | | | | |

**Supplementary table 7, Multivariate model Haematocrit threshold weighted exposure <26% >37%**

| **Variable** | **Adjusted odds ratio (95% CI)** | **p-value** | **Marginal effects estimation (95% CI)** | **p-value** |
| --- | --- | --- | --- | --- |
| Low threshold weighted exposure HCT <26% | 1.004 (1.002-1.006) | < 0.001 | 0.0004 (0.0002-0.0007) | < 0.001 |
| High threshold weighted exposure HCT >37% | 1.004 (1-1.008) | 0.06 | 0.0004 (-0.00001-0.0008) | 0.06 |
| Aortic cross‑clamp time | 1.012 (1.008-1.015) | < 0.001 | 0.0013 (0.0009-0.0017) | < 0.001 |
| Preoperative haemoglobin |  |  |  |  |
| Hb >120 g/L | Reference |  |  |  |
| Hb 100-119 g/L | 1.407 (0.983-1.994) | 0.06 | 0.04 (-0.0046-0.085) | 0.08 |
| Hb <100 g/L | 2.72 (1.021-7.07) | 0.042 | 0.14 (-0.025-0.305) | 0.10 |
| Age |  |  |  |  |
| Age <65 years | Reference |  |  |  |
| Age 65–74 years | 3.02 (2.04-4.57) | < 0.001 | 0.09 (0.063-0.117) | < 0.001 |
| Age ≥75 years | 5.23 (3.38-8.25) | < 0.001 | 0.161 (0.122-0.2) | < 0.001 |
| Male sex | 0.867 (0.662-1.141) | 0.30 | -0.016 (-0.047-0.015) | 0.31 |
| BMI |  |  |  |  |
| BMI <20 kg/m² | 0.809 (0.379-1.583) | 0.56 | -0.022 (-0.09-0.047) | 0.53 |
| BMI 20–24.9 kg/m² | 0.774 (0.582-1.024) | 0.08 | -0.026 (-0.054-0.002) | 0.07 |
| BMI 25–29.9 kg/m² | Reference |  |  |  |
| BMI 30–34.9 kg/m² | 1.33 (0.993-1.774) | 0.05 | 0.034 (-0.0016-0.069) | 0.06 |
| BMI ≥35 kg/m² | 1.957 (1.232-3.05) | 0.0037 | 0.087 (0.021-0.154) | 0.010 |
| Smoking status |  |  |  |  |
| Never smoked | Reference |  |  |  |
| Former smoker | 0.932 (0.734-1.184) | 0.56 | -0.0079 (-0.035-0.019) | 0.56 |
| Current smoker | 0.645 (0.417-0.975) | 0.042 | -0.044 (-0.084--0.0048) | 0.028 |
| NYHA class |  |  |  |  |
| NYHA I | Reference |  |  |  |
| NYHA II | 0.658 (0.481-0.902) | 0.0090 | -0.044 (-0.078--0.0094) | 0.012 |
| NYHA III | 1.16 (0.822-1.64) | 0.40 | 0.018 (-0.024-0.06) | 0.40 |
| NYHA IV | 1.689 (0.956-2.93) | 0.07 | 0.071 (-0.011-0.153) | 0.09 |
| Diabetes | 1.168 (0.892-1.522) | 0.25 | 0.017 (-0.013-0.048) | 0.27 |
| Peripheral vascular disease | 1.474 (0.93-2.29) | 0.09 | 0.047 (-0.012-0.106) | 0.12 |
| Hypertension | 1.445 (1.101-1.906) | 0.0085 | 0.039 (0.011-0.066) | 0.0059 |
| Glomerular filtration rate |  |  |  |  |
| eGFR <30 mL/min/1.73 m2 | 2.25 (1.268-3.95) | 0.0050 | 0.112 (0.02-0.203) | 0.017 |
| eGFR 30–59.9 mL/min/1.73 m2 | 0.646 (0.489-0.847) | 0.0018 | -0.043 (-0.069--0.017) | < 0.001 |
| eGFR 60–89.9 mL/min/1.73 m^2^ | Reference |  |  |  |
| eGFR ≥90 mL/min/1.73 m2 | 2.84 (1.793-4.45) | < 0.001 | 0.151 (0.074-0.228) | < 0.001 |
| Critical preoperative event | 2.32 (0.873-6.18) | 0.09 | 0.115 (-0.045-0.275) | 0.16 |
| Left ventricular ejection fraction |  |  |  |  |
| LVEF Good >50% | Reference |  |  |  |
| LVEF Fair 30-49% | 1.882 (1.457-2.43) | < 0.001 | 0.075 (0.042-0.107) | < 0.001 |
| LVEF Poor <30% | 2.45 (1.538-3.84) | < 0.001 | 0.114 (0.044-0.183) | 0.0013 |
| Operative priority |  |  |  |  |
| Elective | Reference |  |  |  |
| Urgent | 0.879 (0.667-1.157) | 0.36 | -0.014 (-0.043-0.015) | 0.35 |
| Emergency/salvage | 1.378 (0.728-2.52) | 0.31 | 0.039 (-0.042-0.12) | 0.34 |
| Cardiac procedure |  |  |  |  |
| CABG only | Reference |  |  |  |
| Valve only | 0.919 (0.666-1.269) | 0.61 | -0.0091 (-0.044-0.026) | 0.61 |
| CABG + valve | 0.886 (0.546-1.413) | 0.62 | -0.013 (-0.063-0.037) | 0.61 |
| Other/multiple | 1.247 (0.73-2.08) | 0.41 | 0.026 (-0.038-0.09) | 0.43 |
| Abbreviations: BMI, body mass index; CABG, coronary artery bypass graft; eGFR, estimated glomerular filtration rate; Hb, haemoglobin; HCT, haematocrit; LVEF, left ventricular ejection fraction; MAP, mean arterial pressure NYHA, New York Heart Association. | | | | |

**Supplementary table 8, Multivariate model Haematocrit threshold weighted exposure <25% >37%**

| **Variable** | **Adjusted odds ratio (95% CI)** | **p-value** | **Marginal effects estimation (95% CI)** | **p-value** |
| --- | --- | --- | --- | --- |
| Low threshold weighted exposure HCT <25% | 1.006 (1.002-1.009) | < 0.001 | 0.0006 (0.0003-0.0009) | < 0.001 |
| High threshold weighted exposure HCT >37% | 1.004 (1-1.008) | 0.06 | 0.0004 (-0.00002-0.0008) | 0.06 |
| Preoperative haemoglobin |  |  |  |  |
| Hb >120 g/L | Reference |  |  |  |
| Hb 100-119 g/L | 1.431 (1.004-2.02) | 0.044 | 0.043 (-0.0023-0.087) | 0.06 |
| Hb <100 g/L | 2.77 (1.043-7.19) | 0.038 | 0.143 (-0.022-0.308) | 0.09 |
| Aortic cross‑clamp time | 1.012 (1.009-1.015) | < 0.001 | 0.0013 (0.001-0.0017) | < 0.001 |
| Age |  |  |  |  |
| Age <65 years | Reference |  |  |  |
| Age 65–74 years | 3.04 (2.05-4.59) | < 0.001 | 0.09 (0.063-0.117) | < 0.001 |
| Age ≥75 years | 5.25 (3.39-8.28) | < 0.001 | 0.161 (0.123-0.2) | < 0.001 |
| Male sex | 0.858 (0.656-1.128) | 0.27 | -0.017 (-0.048-0.014) | 0.28 |
| BMI |  |  |  |  |
| BMI <20 kg/m² | 0.798 (0.374-1.563) | 0.53 | -0.023 (-0.091-0.045) | 0.51 |
| BMI 20–24.9 kg/m² | 0.779 (0.586-1.03) | 0.08 | -0.025 (-0.053-0.0026) | 0.08 |
| BMI 25–29.9 kg/m² | Reference |  |  |  |
| BMI 30–34.9 kg/m² | 1.326 (0.99-1.769) | 0.06 | 0.033 (-0.0019-0.068) | 0.06 |
| BMI ≥35 kg/m² | 1.95 (1.228-3.04) | 0.0038 | 0.087 (0.02-0.153) | 0.010 |
| Smoking status |  |  |  |  |
| Never smoked | Reference |  |  |  |
| Former smoker | 0.931 (0.733-1.184) | 0.56 | -0.008 (-0.035-0.019) | 0.56 |
| Current smoker | 0.644 (0.417-0.975) | 0.042 | -0.044 (-0.084--0.0048) | 0.028 |
| NYHA class |  |  |  |  |
| NYHA I | Reference |  |  |  |
| NYHA II | 0.657 (0.48-0.9) | 0.0087 | -0.044 (-0.078--0.0096) | 0.012 |
| NYHA III | 1.16 (0.822-1.64) | 0.40 | 0.018 (-0.024-0.061) | 0.40 |
| NYHA IV | 1.695 (0.96-2.95) | 0.06 | 0.072 (-0.011-0.154) | 0.09 |
| Diabetes | 1.167 (0.891-1.521) | 0.26 | 0.017 (-0.013-0.048) | 0.27 |
| Peripheral vascular disease | 1.461 (0.921-2.27) | 0.10 | 0.046 (-0.013-0.105) | 0.13 |
| Hypertension | 1.448 (1.104-1.911) | 0.0081 | 0.039 (0.011-0.066) | 0.0056 |
| Glomerular filtration rate |  |  |  |  |
| eGFR <30 mL/min/1.73 m2 | 2.29 (1.287-4.01) | 0.0042 | 0.114 (0.022-0.206) | 0.015 |
| eGFR 30–59.9 mL/min/1.73 m2 | 0.647 (0.49-0.849) | 0.0019 | -0.043 (-0.069--0.017) | 0.0010 |
| eGFR 60–89.9 mL/min/1.73 m^2^ | Reference |  |  |  |
| eGFR ≥90 mL/min/1.73 m2 | 2.83 (1.791-4.44) | < 0.001 | 0.151 (0.074-0.227) | < 0.001 |
| Critical preoperative event | 2.3 (0.864-6.13) | 0.09 | 0.113 (-0.046-0.273) | 0.16 |
| Left ventricular ejection fraction |  |  |  |  |
| LVEF Good >50% | Reference |  |  |  |
| LVEF Fair 30-49% | 1.884 (1.458-2.43) | < 0.001 | 0.075 (0.042-0.108) | < 0.001 |
| LVEF Poor <30% | 2.45 (1.537-3.83) | < 0.001 | 0.114 (0.044-0.183) | 0.0013 |
| Operative priority |  |  |  |  |
| Elective | Reference |  |  |  |
| Urgent | 0.88 (0.667-1.158) | 0.36 | -0.014 (-0.043-0.016) | 0.36 |
| Emergency/salvage | 1.38 (0.729-2.52) | 0.31 | 0.039 (-0.042-0.121) | 0.34 |
| Cardiac procedure |  |  |  |  |
| CABG only | Reference |  |  |  |
| Valve only | 0.919 (0.665-1.268) | 0.61 | -0.0092 (-0.044-0.026) | 0.60 |
| CABG + valve | 0.886 (0.547-1.413) | 0.62 | -0.013 (-0.063-0.037) | 0.61 |
| Other/multiple | 1.242 (0.728-2.07) | 0.42 | 0.026 (-0.038-0.09) | 0.43 |
| Abbreviations: BMI, body mass index; CABG, coronary artery bypass graft; eGFR, estimated glomerular filtration rate; Hb, haemoglobin; HCT, haematocrit; LVEF, left ventricular ejection fraction; MAP, mean arterial pressure NYHA, New York Heart Association. | | | | |

**Supplementary table 9, Multivariate model Haematocrit threshold weighted exposure <27% >38%**

| **Variable** | **Adjusted odds ratio (95% CI)** | **p-value** | **Marginal effects estimation (95% CI)** | **p-value** |
| --- | --- | --- | --- | --- |
| Low threshold weighted exposure HCT <27% | 1.003 (1.001-1.004) | < 0.001 | 0.0003 (0.0001-0.0005) | < 0.001 |
| High threshold weighted exposure HCT >38% | 1.006 (1.001-1.012) | 0.016 | 0.0007 (0.0001-0.0013) | 0.015 |
| Aortic cross‑clamp time | 1.012 (1.008-1.015) | < 0.001 | 0.0013 (0.0009-0.0016) | < 0.001 |
| Preoperative haemoglobin |  |  |  |  |
| Hb >120 g/L | Reference |  |  |  |
| Hb 100-119 g/L | 1.387 (0.965-1.973) | 0.07 | 0.039 (-0.0066-0.084) | 0.09 |
| Hb <100 g/L | 2.68 (1.008-6.99) | 0.044 | 0.138 (-0.026-0.302) | 0.10 |
| Age |  |  |  |  |
| Age <65 years | Reference |  |  |  |
| Age 65–74 years | 3.05 (2.06-4.61) | < 0.001 | 0.09 (0.063-0.117) | < 0.001 |
| Age ≥75 years | 5.27 (3.4-8.33) | < 0.001 | 0.162 (0.123-0.2) | < 0.001 |
| Male sex | 0.876 (0.668-1.154) | 0.34 | -0.015 (-0.046-0.016) | 0.35 |
| BMI |  |  |  |  |
| BMI <20 kg/m² | 0.816 (0.382-1.597) | 0.57 | -0.021 (-0.089-0.048) | 0.55 |
| BMI 20–24.9 kg/m² | 0.771 (0.58-1.021) | 0.07 | -0.026 (-0.054-0.0017) | 0.07 |
| BMI 25–29.9 kg/m² | Reference |  |  |  |
| BMI 30–34.9 kg/m² | 1.341 (1.001-1.789) | 0.048 | 0.035 (-0.0007-0.07) | 0.05 |
| BMI ≥35 kg/m² | 1.961 (1.233-3.06) | 0.0036 | 0.088 (0.021-0.154) | 0.010 |
| Smoking status |  |  |  |  |
| Never smoked | Reference |  |  |  |
| Former smoker | 0.933 (0.735-1.186) | 0.57 | -0.0078 (-0.035-0.019) | 0.57 |
| Current smoker | 0.647 (0.419-0.979) | 0.044 | -0.044 (-0.084--0.0044) | 0.029 |
| NYHA class |  |  |  |  |
| NYHA I | Reference |  |  |  |
| NYHA II | 0.657 (0.48-0.9) | 0.0087 | -0.044 (-0.078--0.0096) | 0.012 |
| NYHA III | 1.158 (0.821-1.637) | 0.40 | 0.018 (-0.024-0.06) | 0.40 |
| NYHA IV | 1.671 (0.946-2.9) | 0.07 | 0.07 (-0.012-0.151) | 0.10 |
| Diabetes | 1.165 (0.889-1.518) | 0.26 | 0.017 (-0.014-0.048) | 0.27 |
| Peripheral vascular disease | 1.48 (0.934-2.29) | 0.09 | 0.047 (-0.012-0.107) | 0.12 |
| Hypertension | 1.435 (1.095-1.894) | 0.0097 | 0.038 (0.01-0.065) | 0.0069 |
| Glomerular filtration rate |  |  |  |  |
| eGFR <30 mL/min/1.73 m2 | 2.23 (1.256-3.92) | 0.0055 | 0.11 (0.019-0.202) | 0.018 |
| eGFR 30–59.9 mL/min/1.73 m2 | 0.645 (0.489-0.846) | 0.0017 | -0.043 (-0.069--0.018) | < 0.001 |
| eGFR 60–89.9 mL/min/1.73 m^2^ | Reference |  |  |  |
| eGFR ≥90 mL/min/1.73 m2 | 2.85 (1.8-4.47) | < 0.001 | 0.152 (0.075-0.229) | < 0.001 |
| Critical preoperative event | 2.34 (0.882-6.24) | 0.09 | 0.117 (-0.044-0.277) | 0.15 |
| Left ventricular ejection fraction |  |  |  |  |
| LVEF Good >50% | Reference |  |  |  |
| LVEF Fair 30-49% | 1.88 (1.455-2.42) | < 0.001 | 0.075 (0.042-0.107) | < 0.001 |
| LVEF Poor <30% | 2.45 (1.537-3.84) | < 0.001 | 0.114 (0.044-0.183) | 0.0013 |
| Operative priority |  |  |  |  |
| Elective | Reference |  |  |  |
| Urgent | 0.878 (0.666-1.156) | 0.36 | -0.014 (-0.043-0.015) | 0.35 |
| Emergency/salvage | 1.375 (0.727-2.51) | 0.31 | 0.039 (-0.042-0.12) | 0.35 |
| Cardiac procedure |  |  |  |  |
| CABG only | Reference |  |  |  |
| Valve only | 0.917 (0.664-1.266) | 0.60 | -0.0094 (-0.044-0.025) | 0.60 |
| CABG + valve | 0.878 (0.541-1.401) | 0.59 | -0.014 (-0.064-0.036) | 0.58 |
| Other/multiple | 1.255 (0.735-2.09) | 0.39 | 0.027 (-0.038-0.091) | 0.41 |
| Abbreviations: BMI, body mass index; CABG, coronary artery bypass graft; eGFR, estimated glomerular filtration rate; Hb, haemoglobin; HCT, haematocrit; LVEF, left ventricular ejection fraction; MAP, mean arterial pressure NYHA, New York Heart Association. | | | | |

**Supplementary table 10, Multivariate model Haematocrit threshold weighted exposure <26% >38%**

| **Variable** | **Adjusted odds ratio (95% CI)** | **p-value** | **Marginal effects estimation (95% CI)** | **p-value** |
| --- | --- | --- | --- | --- |
| Low threshold weighted exposure HCT <26% | 1.004 (1.002-1.006) | < 0.001 | 0.0004 (0.0002-0.0007) | < 0.001 |
| High threshold weighted exposure HCT >38% | 1.006 (1.001-1.012) | 0.017 | 0.0007 (0.0001-0.0013) | 0.017 |
| Aortic cross‑clamp time | 1.012 (1.008-1.015) | < 0.001 | 0.0013 (0.0009-0.0017) | < 0.001 |
| Preoperative haemoglobin |  |  |  |  |
| Hb >120 g/L | Reference |  |  |  |
| Hb 100-119 g/L | 1.41 (0.985-1.998) | 0.06 | 0.041 (-0.0044-0.086) | 0.08 |
| Hb <100 g/L | 2.73 (1.025-7.1) | 0.041 | 0.14 (-0.024-0.305) | 0.09 |
| Age |  |  |  |  |
| Age <65 years | Reference |  |  |  |
| Age 65–74 years | 3.05 (2.06-4.61) | < 0.001 | 0.09 (0.063-0.117) | < 0.001 |
| Age ≥75 years | 5.27 (3.4-8.33) | < 0.001 | 0.162 (0.123-0.2) | < 0.001 |
| Male sex | 0.868 (0.663-1.142) | 0.31 | -0.016 (-0.047-0.015) | 0.32 |
| BMI |  |  |  |  |
| BMI <20 kg/m² | 0.811 (0.38-1.588) | 0.56 | -0.021 (-0.09-0.047) | 0.54 |
| BMI 20–24.9 kg/m² | 0.776 (0.584-1.027) | 0.08 | -0.025 (-0.053-0.0023) | 0.07 |
| BMI 25–29.9 kg/m² | Reference |  |  |  |
| BMI 30–34.9 kg/m² | 1.335 (0.996-1.781) | 0.05 | 0.034 (-0.0012-0.069) | 0.06 |
| BMI ≥35 kg/m² | 1.952 (1.228-3.05) | 0.0038 | 0.087 (0.02-0.154) | 0.010 |
| Smoking status |  |  |  |  |
| Never smoked | Reference |  |  |  |
| Former smoker | 0.933 (0.735-1.186) | 0.57 | -0.0078 (-0.035-0.019) | 0.57 |
| Current smoker | 0.646 (0.418-0.978) | 0.044 | -0.044 (-0.084--0.0045) | 0.029 |
| NYHA class |  |  |  |  |
| NYHA I | Reference |  |  |  |
| NYHA II | 0.657 (0.48-0.9) | 0.0087 | -0.044 (-0.078--0.0096) | 0.012 |
| NYHA III | 1.157 (0.82-1.636) | 0.41 | 0.018 (-0.024-0.06) | 0.40 |
| NYHA IV | 1.684 (0.954-2.93) | 0.07 | 0.071 (-0.011-0.153) | 0.09 |
| Diabetes | 1.166 (0.891-1.52) | 0.26 | 0.017 (-0.013-0.048) | 0.27 |
| Peripheral vascular disease | 1.47 (0.927-2.28) | 0.09 | 0.046 (-0.013-0.106) | 0.12 |
| Hypertension | 1.441 (1.099-1.902) | 0.0090 | 0.038 (0.011-0.066) | 0.0063 |
| Glomerular filtration rate |  |  |  |  |
| eGFR <30 mL/min/1.73 m2 | 2.25 (1.267-3.95) | 0.0050 | 0.112 (0.02-0.203) | 0.017 |
| eGFR 30–59.9 mL/min/1.73 m2 | 0.645 (0.489-0.847) | 0.0018 | -0.043 (-0.069--0.018) | < 0.001 |
| eGFR 60–89.9 mL/min/1.73 m^2^ | Reference |  |  |  |
| eGFR ≥90 mL/min/1.73 m2 | 2.84 (1.792-4.45) | < 0.001 | 0.151 (0.074-0.228) | < 0.001 |
| Critical preoperative event | 2.32 (0.874-6.2) | 0.09 | 0.115 (-0.045-0.275) | 0.16 |
| Left ventricular ejection fraction |  |  |  |  |
| LVEF Good >50% | Reference |  |  |  |
| LVEF Fair 30-49% | 1.883 (1.457-2.43) | < 0.001 | 0.075 (0.042-0.107) | < 0.001 |
| LVEF Poor <30% | 2.44 (1.533-3.83) | < 0.001 | 0.113 (0.044-0.183) | 0.0014 |
| Operative priority |  |  |  |  |
| Elective | Reference |  |  |  |
| Urgent | 0.881 (0.668-1.159) | 0.37 | -0.014 (-0.043-0.016) | 0.36 |
| Emergency/salvage | 1.378 (0.728-2.52) | 0.31 | 0.039 (-0.042-0.12) | 0.35 |
| Cardiac procedure |  |  |  |  |
| CABG only | Reference |  |  |  |
| Valve only | 0.917 (0.664-1.266) | 0.60 | -0.0094 (-0.044-0.025) | 0.60 |
| CABG + valve | 0.881 (0.543-1.406) | 0.60 | -0.014 (-0.063-0.036) | 0.59 |
| Other/multiple | 1.248 (0.731-2.08) | 0.41 | 0.026 (-0.038-0.09) | 0.42 |
| Abbreviations: BMI, body mass index; CABG, coronary artery bypass graft; eGFR, estimated glomerular filtration rate; Hb, haemoglobin; HCT, haematocrit; LVEF, left ventricular ejection fraction; MAP, mean arterial pressure NYHA, New York Heart Association. | | | | |

**Supplementary table 11, Multivariate model Haematocrit threshold weighted exposure <25% >38%**

| **Variable** | **Adjusted odds ratio (95% CI)** | **p-value** | **Marginal effects estimation (95% CI)** | **p-value** |
| --- | --- | --- | --- | --- |
| Low threshold weighted exposure HCT <25% | 1.006 (1.002-1.009) | < 0.001 | 0.0006 (0.0003-0.0009) | < 0.001 |
| High threshold weighted exposure HCT >38% | 1.006 (1.001-1.011) | 0.019 | 0.0007 (0.0001-0.0012) | 0.019 |
| Preoperative haemoglobin |  |  |  |  |
| Hb >120 g/L | Reference |  |  |  |
| Hb 100-119 g/L | 1.434 (1.006-2.02) | 0.043 | 0.043 (-0.0021-0.088) | 0.06 |
| Hb <100 g/L | 2.78 (1.048-7.22) | 0.037 | 0.144 (-0.022-0.309) | 0.09 |
| Aortic cross‑clamp time | 1.012 (1.009-1.015) | < 0.001 | 0.0013 (0.001-0.0017) | < 0.001 |
| Age |  |  |  |  |
| Age <65 years | Reference |  |  |  |
| Age 65–74 years | 3.06 (2.07-4.63) | < 0.001 | 0.09 (0.063-0.117) | < 0.001 |
| Age ≥75 years | 5.29 (3.41-8.36) | < 0.001 | 0.162 (0.123-0.2) | < 0.001 |
| Male sex | 0.859 (0.657-1.128) | 0.27 | -0.017 (-0.048-0.014) | 0.28 |
| BMI |  |  |  |  |
| BMI <20 kg/m² | 0.801 (0.375-1.569) | 0.54 | -0.023 (-0.09-0.045) | 0.51 |
| BMI 20–24.9 kg/m² | 0.781 (0.587-1.033) | 0.09 | -0.025 (-0.053-0.0029) | 0.08 |
| BMI 25–29.9 kg/m² | Reference |  |  |  |
| BMI 30–34.9 kg/m² | 1.331 (0.994-1.776) | 0.05 | 0.034 (-0.0015-0.069) | 0.06 |
| BMI ≥35 kg/m² | 1.945 (1.224-3.04) | 0.0040 | 0.086 (0.02-0.153) | 0.011 |
| Smoking status |  |  |  |  |
| Never smoked | Reference |  |  |  |
| Former smoker | 0.932 (0.734-1.185) | 0.57 | -0.0079 (-0.035-0.019) | 0.57 |
| Current smoker | 0.646 (0.418-0.978) | 0.043 | -0.044 (-0.084--0.0045) | 0.029 |
| NYHA class |  |  |  |  |
| NYHA I | Reference |  |  |  |
| NYHA II | 0.655 (0.479-0.899) | 0.0084 | -0.044 (-0.078--0.0098) | 0.012 |
| NYHA III | 1.158 (0.82-1.637) | 0.41 | 0.018 (-0.024-0.06) | 0.40 |
| NYHA IV | 1.691 (0.957-2.94) | 0.07 | 0.071 (-0.011-0.154) | 0.09 |
| Diabetes | 1.165 (0.89-1.519) | 0.26 | 0.017 (-0.014-0.048) | 0.27 |
| Peripheral vascular disease | 1.457 (0.918-2.26) | 0.10 | 0.045 (-0.014-0.104) | 0.13 |
| Hypertension | 1.444 (1.101-1.907) | 0.0086 | 0.039 (0.011-0.066) | 0.0059 |
| Glomerular filtration rate |  |  |  |  |
| eGFR <30 mL/min/1.73 m2 | 2.29 (1.287-4.01) | 0.0042 | 0.114 (0.022-0.206) | 0.015 |
| eGFR 30–59.9 mL/min/1.73 m2 | 0.646 (0.489-0.848) | 0.0018 | -0.043 (-0.069--0.017) | < 0.001 |
| eGFR 60–89.9 mL/min/1.73 m^2^ | Reference |  |  |  |
| eGFR ≥90 mL/min/1.73 m2 | 2.83 (1.79-4.44) | < 0.001 | 0.151 (0.074-0.227) | < 0.001 |
| Critical preoperative event | 2.3 (0.865-6.15) | 0.09 | 0.114 (-0.046-0.273) | 0.16 |
| Left ventricular ejection fraction |  |  |  |  |
| LVEF Good >50% | Reference |  |  |  |
| LVEF Fair 30-49% | 1.885 (1.459-2.43) | < 0.001 | 0.075 (0.042-0.108) | < 0.001 |
| LVEF Poor <30% | 2.44 (1.533-3.83) | < 0.001 | 0.113 (0.044-0.182) | 0.0014 |
| Operative priority |  |  |  |  |
| Elective | Reference |  |  |  |
| Urgent | 0.882 (0.668-1.16) | 0.37 | -0.014 (-0.043-0.016) | 0.37 |
| Emergency/salvage | 1.38 (0.728-2.52) | 0.31 | 0.039 (-0.042-0.12) | 0.34 |
| Cardiac procedure |  |  |  |  |
| CABG only | Reference |  |  |  |
| Valve only | 0.916 (0.664-1.265) | 0.59 | -0.0095 (-0.044-0.025) | 0.59 |
| CABG + valve | 0.881 (0.543-1.406) | 0.60 | -0.014 (-0.063-0.036) | 0.59 |
| Other/multiple | 1.243 (0.728-2.08) | 0.41 | 0.026 (-0.038-0.09) | 0.43 |
| Abbreviations: BMI, body mass index; CABG, coronary artery bypass graft; eGFR, estimated glomerular filtration rate; Hb, haemoglobin; HCT, haematocrit; LVEF, left ventricular ejection fraction; MAP, mean arterial pressure NYHA, New York Heart Association. | | | | |

**Supplementary table 12, Multivariate model Haematocrit threshold weighted exposure <27% >39%**

| **Variable** | **Adjusted odds ratio (95% CI)** | **p-value** | **Marginal effects estimation (95% CI)** | **p-value** |
| --- | --- | --- | --- | --- |
| Low threshold weighted exposure HCT <27% | 1.003 (1.001-1.004) | < 0.001 | 0.0003 (0.0001-0.0005) | < 0.001 |
| High threshold weighted exposure HCT >39% | 1.01 (1.002-1.017) | 0.011 | 0.0011 (0.0002-0.0019) | 0.010 |
| Preoperative haemoglobin |  |  |  |  |
| Hb >120 g/L | Reference |  |  |  |
| Hb 100-119 g/L | 1.387 (0.965-1.972) | 0.07 | 0.039 (-0.0066-0.084) | 0.09 |
| Hb <100 g/L | 2.69 (1.008-7) | 0.044 | 0.138 (-0.026-0.302) | 0.10 |
| Aortic cross‑clamp time | 1.012 (1.008-1.015) | < 0.001 | 0.0013 (0.0009-0.0016) | < 0.001 |
| Age |  |  |  |  |
| Age <65 years | Reference |  |  |  |
| Age 65–74 years | 3.06 (2.06-4.62) | < 0.001 | 0.09 (0.063-0.117) | < 0.001 |
| Age ≥75 years | 5.28 (3.41-8.35) | < 0.001 | 0.161 (0.123-0.2) | < 0.001 |
| Male sex | 0.88 (0.671-1.158) | 0.36 | -0.014 (-0.045-0.017) | 0.37 |
| BMI |  |  |  |  |
| BMI <20 kg/m² | 0.816 (0.382-1.598) | 0.57 | -0.021 (-0.089-0.048) | 0.55 |
| BMI 20–24.9 kg/m² | 0.772 (0.58-1.021) | 0.07 | -0.026 (-0.054-0.0017) | 0.07 |
| BMI 25–29.9 kg/m² | Reference |  |  |  |
| BMI 30–34.9 kg/m² | 1.344 (1.003-1.793) | 0.046 | 0.035 (-0.0005-0.07) | 0.05 |
| BMI ≥35 kg/m² | 1.961 (1.232-3.06) | 0.0037 | 0.088 (0.021-0.154) | 0.010 |
| Smoking status |  |  |  |  |
| Never smoked | Reference |  |  |  |
| Former smoker | 0.933 (0.735-1.186) | 0.57 | -0.0078 (-0.035-0.019) | 0.57 |
| Current smoker | 0.649 (0.42-0.982) | 0.046 | -0.044 (-0.083--0.004) | 0.031 |
| NYHA class |  |  |  |  |
| NYHA I | Reference |  |  |  |
| NYHA II | 0.657 (0.48-0.9) | 0.0086 | -0.044 (-0.078--0.0097) | 0.012 |
| NYHA III | 1.156 (0.819-1.635) | 0.41 | 0.018 (-0.024-0.06) | 0.41 |
| NYHA IV | 1.664 (0.942-2.89) | 0.07 | 0.069 (-0.013-0.151) | 0.10 |
| Diabetes | 1.164 (0.889-1.517) | 0.27 | 0.017 (-0.014-0.048) | 0.28 |
| Peripheral vascular disease | 1.476 (0.931-2.29) | 0.09 | 0.047 (-0.012-0.106) | 0.12 |
| Hypertension | 1.435 (1.094-1.894) | 0.0098 | 0.038 (0.01-0.065) | 0.0069 |
| Glomerular filtration rate |  |  |  |  |
| eGFR <30 mL/min/1.73 m2 | 2.24 (1.258-3.92) | 0.0054 | 0.111 (0.019-0.202) | 0.017 |
| eGFR 30–59.9 mL/min/1.73 m2 | 0.645 (0.489-0.846) | 0.0017 | -0.043 (-0.069--0.018) | < 0.001 |
| eGFR 60–89.9 mL/min/1.73 m^2^ | Reference |  |  |  |
| eGFR ≥90 mL/min/1.73 m2 | 2.85 (1.803-4.48) | < 0.001 | 0.152 (0.075-0.229) | < 0.001 |
| Critical preoperative event | 2.35 (0.885-6.26) | 0.09 | 0.117 (-0.043-0.277) | 0.15 |
| Left ventricular ejection fraction |  |  |  |  |
| LVEF Good >50% | Reference |  |  |  |
| LVEF Fair 30-49% | 1.881 (1.455-2.42) | < 0.001 | 0.075 (0.042-0.107) | < 0.001 |
| LVEF Poor <30% | 2.44 (1.534-3.83) | < 0.001 | 0.113 (0.044-0.183) | 0.0014 |
| Operative priority |  |  |  |  |
| Elective | Reference |  |  |  |
| Urgent | 0.879 (0.666-1.157) | 0.36 | -0.014 (-0.043-0.015) | 0.35 |
| Emergency/salvage | 1.375 (0.727-2.51) | 0.31 | 0.039 (-0.042-0.12) | 0.35 |
| Cardiac procedure |  |  |  |  |
| CABG only | Reference |  |  |  |
| Valve only | 0.919 (0.666-1.268) | 0.61 | -0.0092 (-0.044-0.026) | 0.61 |
| CABG + valve | 0.875 (0.54-1.398) | 0.58 | -0.014 (-0.064-0.035) | 0.57 |
| Other/multiple | 1.258 (0.737-2.1) | 0.39 | 0.027 (-0.037-0.092) | 0.41 |
| Abbreviations: BMI, body mass index; CABG, coronary artery bypass graft; eGFR, estimated glomerular filtration rate; Hb, haemoglobin; HCT, haematocrit; LVEF, left ventricular ejection fraction; MAP, mean arterial pressure NYHA, New York Heart Association. | | | | |

**Supplementary table 13, Multivariate model Haematocrit threshold weighted exposure <26% >39%**

| **Variable** | **Adjusted odds ratio (95% CI)** | **p-value** | **Marginal effects estimation (95% CI)** | **p-value** |
| --- | --- | --- | --- | --- |
| Low threshold weighted exposure HCT <26% | 1.004 (1.002-1.006) | < 0.001 | 0.0004 (0.0002-0.0007) | < 0.001 |
| High threshold weighted exposure HCT >39% | 1.01 (1.002-1.017) | 0.012 | 0.001 (0.0002-0.0018) | 0.012 |
| Preoperative haemoglobin |  |  |  |  |
| Hb >120 g/L | Reference |  |  |  |
| Hb 100-119 g/L | 1.41 (0.985-1.997) | 0.06 | 0.041 (-0.0044-0.086) | 0.08 |
| Hb <100 g/L | 2.73 (1.026-7.11) | 0.041 | 0.14 (-0.024-0.305) | 0.09 |
| Aortic cross‑clamp time | 1.012 (1.008-1.015) | < 0.001 | 0.0013 (0.0009-0.0017) | < 0.001 |
| Age |  |  |  |  |
| Age <65 years | Reference |  |  |  |
| Age 65–74 years | 3.05 (2.06-4.62) | < 0.001 | 0.09 (0.063-0.117) | < 0.001 |
| Age ≥75 years | 5.28 (3.41-8.34) | < 0.001 | 0.161 (0.123-0.2) | < 0.001 |
| Male sex | 0.872 (0.666-1.146) | 0.32 | -0.015 (-0.046-0.016) | 0.33 |
| BMI |  |  |  |  |
| BMI <20 kg/m² | 0.811 (0.38-1.588) | 0.56 | -0.021 (-0.09-0.047) | 0.54 |
| BMI 20–24.9 kg/m² | 0.776 (0.584-1.027) | 0.08 | -0.025 (-0.053-0.0023) | 0.07 |
| BMI 25–29.9 kg/m² | Reference |  |  |  |
| BMI 30–34.9 kg/m² | 1.338 (0.998-1.785) | 0.049 | 0.034 (-0.001-0.069) | 0.06 |
| BMI ≥35 kg/m² | 1.952 (1.227-3.05) | 0.0039 | 0.087 (0.02-0.153) | 0.011 |
| Smoking status |  |  |  |  |
| Never smoked | Reference |  |  |  |
| Former smoker | 0.933 (0.735-1.186) | 0.57 | -0.0077 (-0.035-0.019) | 0.57 |
| Current smoker | 0.649 (0.42-0.981) | 0.045 | -0.044 (-0.083--0.0041) | 0.031 |
| NYHA class |  |  |  |  |
| NYHA I | Reference |  |  |  |
| NYHA II | 0.657 (0.48-0.9) | 0.0086 | -0.044 (-0.078--0.0097) | 0.012 |
| NYHA III | 1.156 (0.819-1.634) | 0.41 | 0.018 (-0.024-0.06) | 0.41 |
| NYHA IV | 1.677 (0.949-2.91) | 0.07 | 0.07 (-0.012-0.152) | 0.09 |
| Diabetes | 1.165 (0.89-1.519) | 0.26 | 0.017 (-0.014-0.048) | 0.27 |
| Peripheral vascular disease | 1.466 (0.924-2.28) | 0.10 | 0.046 (-0.013-0.105) | 0.13 |
| Hypertension | 1.44 (1.098-1.901) | 0.0091 | 0.038 (0.011-0.066) | 0.0063 |
| Glomerular filtration rate |  |  |  |  |
| eGFR <30 mL/min/1.73 m2 | 2.26 (1.27-3.96) | 0.0049 | 0.112 (0.021-0.203) | 0.016 |
| eGFR 30–59.9 mL/min/1.73 m2 | 0.645 (0.489-0.847) | 0.0018 | -0.043 (-0.069--0.018) | < 0.001 |
| eGFR 60–89.9 mL/min/1.73 m^2^ | Reference |  |  |  |
| eGFR ≥90 mL/min/1.73 m2 | 2.84 (1.795-4.46) | < 0.001 | 0.151 (0.074-0.228) | < 0.001 |
| Critical preoperative event | 2.33 (0.877-6.22) | 0.09 | 0.116 (-0.044-0.276) | 0.16 |
| Left ventricular ejection fraction |  |  |  |  |
| LVEF Good >50% | Reference |  |  |  |
| LVEF Fair 30-49% | 1.884 (1.458-2.43) | < 0.001 | 0.075 (0.042-0.107) | < 0.001 |
| LVEF Poor <30% | 2.44 (1.53-3.82) | < 0.001 | 0.113 (0.044-0.182) | 0.0014 |
| Operative priority |  |  |  |  |
| Elective | Reference |  |  |  |
| Urgent | 0.881 (0.668-1.16) | 0.37 | -0.014 (-0.043-0.016) | 0.36 |
| Emergency/salvage | 1.377 (0.727-2.52) | 0.31 | 0.039 (-0.042-0.12) | 0.35 |
| Cardiac procedure |  |  |  |  |
| CABG only | Reference |  |  |  |
| Valve only | 0.919 (0.665-1.268) | 0.61 | -0.0092 (-0.044-0.026) | 0.61 |
| CABG + valve | 0.878 (0.541-1.402) | 0.59 | -0.014 (-0.064-0.036) | 0.58 |
| Other/multiple | 1.251 (0.732-2.09) | 0.40 | 0.026 (-0.038-0.091) | 0.42 |
| Abbreviations: BMI, body mass index; CABG, coronary artery bypass graft; eGFR, estimated glomerular filtration rate; Hb, haemoglobin; HCT, haematocrit; LVEF, left ventricular ejection fraction; MAP, mean arterial pressure NYHA, New York Heart Association. | | | | |

**Supplementary table 14, Multivariate model Haematocrit threshold weighted exposure <25% >39%**

| **Variable** | **Adjusted odds ratio (95% CI)** | **p-value** | **Marginal effects estimation (95% CI)** | **p-value** |
| --- | --- | --- | --- | --- |
| Low threshold weighted exposure HCT <25% | 1.006 (1.002-1.009) | < 0.001 | 0.0006 (0.0003-0.0009) | < 0.001 |
| High threshold weighted exposure HCT >39% | 1.009 (1.002-1.017) | 0.013 | 0.001 (0.0002-0.0018) | 0.013 |
| Preoperative haemoglobin |  |  |  |  |
| Hb >120 g/L | Reference |  |  |  |
| Hb 100-119 g/L | 1.434 (1.005-2.02) | 0.043 | 0.043 (-0.0021-0.088) | 0.06 |
| Hb <100 g/L | 2.78 (1.049-7.23) | 0.037 | 0.144 (-0.022-0.309) | 0.09 |
| Aortic cross‑clamp time | 1.012 (1.009-1.015) | < 0.001 | 0.0013 (0.001-0.0017) | < 0.001 |
| Age |  |  |  |  |
| Age <65 years | Reference |  |  |  |
| Age 65–74 years | 3.07 (2.07-4.64) | < 0.001 | 0.09 (0.063-0.117) | < 0.001 |
| Age ≥75 years | 5.3 (3.42-8.38) | < 0.001 | 0.162 (0.123-0.2) | < 0.001 |
| Male sex | 0.863 (0.66-1.133) | 0.28 | -0.016 (-0.047-0.014) | 0.29 |
| BMI |  |  |  |  |
| BMI <20 kg/m² | 0.801 (0.375-1.569) | 0.54 | -0.023 (-0.09-0.045) | 0.52 |
| BMI 20–24.9 kg/m² | 0.781 (0.588-1.033) | 0.09 | -0.025 (-0.053-0.0029) | 0.08 |
| BMI 25–29.9 kg/m² | Reference |  |  |  |
| BMI 30–34.9 kg/m² | 1.334 (0.996-1.78) | 0.05 | 0.034 (-0.0013-0.069) | 0.06 |
| BMI ≥35 kg/m² | 1.945 (1.224-3.04) | 0.0040 | 0.086 (0.02-0.153) | 0.011 |
| Smoking status |  |  |  |  |
| Never smoked | Reference |  |  |  |
| Former smoker | 0.933 (0.734-1.186) | 0.57 | -0.0078 (-0.035-0.019) | 0.57 |
| Current smoker | 0.648 (0.419-0.981) | 0.045 | -0.044 (-0.083--0.0041) | 0.030 |
| NYHA class |  |  |  |  |
| NYHA I | Reference |  |  |  |
| NYHA II | 0.655 (0.479-0.898) | 0.0083 | -0.044 (-0.078--0.0098) | 0.012 |
| NYHA III | 1.156 (0.819-1.635) | 0.41 | 0.018 (-0.024-0.06) | 0.41 |
| NYHA IV | 1.684 (0.953-2.93) | 0.07 | 0.071 (-0.011-0.153) | 0.09 |
| Diabetes | 1.164 (0.889-1.518) | 0.26 | 0.017 (-0.014-0.048) | 0.28 |
| Peripheral vascular disease | 1.453 (0.915-2.26) | 0.10 | 0.045 (-0.014-0.104) | 0.14 |
| Hypertension | 1.444 (1.101-1.906) | 0.0087 | 0.038 (0.011-0.066) | 0.0060 |
| Glomerular filtration rate |  |  |  |  |
| eGFR <30 mL/min/1.73 m2 | 2.29 (1.289-4.01) | 0.0042 | 0.114 (0.023-0.206) | 0.015 |
| eGFR 30–59.9 mL/min/1.73 m2 | 0.646 (0.489-0.848) | 0.0018 | -0.043 (-0.069--0.017) | < 0.001 |
| eGFR 60–89.9 mL/min/1.73 m^2^ | Reference |  |  |  |
| eGFR ≥90 mL/min/1.73 m2 | 2.84 (1.793-4.46) | < 0.001 | 0.151 (0.074-0.228) | < 0.001 |
| Critical preoperative event | 2.31 (0.868-6.17) | 0.09 | 0.114 (-0.045-0.274) | 0.16 |
| Left ventricular ejection fraction |  |  |  |  |
| LVEF Good >50% | Reference |  |  |  |
| LVEF Fair 30-49% | 1.886 (1.459-2.43) | < 0.001 | 0.075 (0.042-0.108) | < 0.001 |
| LVEF Poor <30% | 2.44 (1.53-3.82) | < 0.001 | 0.113 (0.043-0.182) | 0.0014 |
| Operative priority |  |  |  |  |
| Elective | Reference |  |  |  |
| Urgent | 0.882 (0.669-1.161) | 0.37 | -0.013 (-0.043-0.016) | 0.37 |
| Emergency/salvage | 1.379 (0.728-2.52) | 0.31 | 0.039 (-0.042-0.12) | 0.34 |
| Cardiac procedure |  |  |  |  |
| CABG only | Reference |  |  |  |
| Valve only | 0.918 (0.665-1.267) | 0.60 | -0.0093 (-0.044-0.026) | 0.60 |
| CABG + valve | 0.878 (0.541-1.402) | 0.59 | -0.014 (-0.064-0.036) | 0.58 |
| Other/multiple | 1.246 (0.73-2.08) | 0.41 | 0.026 (-0.038-0.09) | 0.43 |
| Abbreviations: BMI, body mass index; CABG, coronary artery bypass graft; eGFR, estimated glomerular filtration rate; Hb, haemoglobin; HCT, haematocrit; LVEF, left ventricular ejection fraction; MAP, mean arterial pressure NYHA, New York Heart Association. | | | | |

**Supplementary table 15,** **Area under the ROC curve and Hosmer-Lemeshow goodness off fit for the haematocrit threshold weighted exposure multivariate model.**

| **Model** | **AUC (95% CI)** | **Hosmer-Lemeshow test** | |
| --- | --- | --- | --- |
|  |  | ***X*^2^** | ***p*-value** |
| Haematocrit threshold weighted exposure <27% >37% | 0.7531 (0.7278-0.7783) | 6.21 | 0.62 |
| Haematocrit threshold weighted exposure <26% >37% | 0.7532 (0.7279-0.7784) | 5.97 | 0.65 |
| Haematocrit threshold weighted exposure <25% >37% | 0.7534 (0.7281-0.7788) | 4.64 | 0.80 |
| Haematocrit threshold weighted exposure <27% >38% | 0.7535 (0.7282-0.7787) | 5.55 | 0.70 |
| Haematocrit threshold weighted exposure <26% >38% | 0.7536 (0.7283-0.7789) | 8.20 | 0.41 |
| Haematocrit threshold weighted exposure <25% >38% | 0.7539 (0.7286-0.7792) | 5.49 | 0.70 |
| Haematocrit threshold weighted exposure <27% >39% | 0.7536 (0.7284-0.7788) | 5.89 | 0.66 |
| Haematocrit threshold weighted exposure <26% >39% | 0.7537 (0.7284-0.779) | 7.13 | 0.52 |
| Haematocrit threshold weighted exposure <25% >39% | 0.754 (0.7287-0.7793) | 5.45 | 0.71 |

**Supplementary table16 Multivariate model Sensitivity analysis**

| **Variable** | **Adjusted odds ratio (95% CI)** | ***p-*value** |
| --- | --- | --- |
| Low threshold weighted exposure HCT <25% | 1.005 (1.001-1.008) per 1 %⋅min increase | 0.0069 |
| High threshold weighted exposure HCT >38% | 1.006 (1-1.011) per 1 %⋅min increase | 0.040 |
| Preoperative haemoglobin |  |  |
| Hb >120 g/L | Reference |  |
| Hb 100-119 g/L | 1.385 (0.911-2.09) | 0.12 |
| Hb <100 g/L | 2.47 (0.803-7.52) | 0.11 |
| Aortic cross‑clamp time | 1.012 (1.008-1.015) per 1⋅min increase | < 0.001 |
| Haematocrit reduction on cardiopulmonary bypass |  |  |
| Restrictive cubic spline 1 | 0.151 (0.02-1.234) per 1 %⋅change | 0.07 |
| Restrictive cubic spline 2 | 0.38 (0.096-1.535) per 1 %⋅change | 0.17 |
| Restrictive cubic spline 3 | 0.047 (0.0005-4.74) per 1 %⋅change | 0.18 |
| Restrictive cubic spline 4 | 0.618 (0.047-7.1) per 1 %⋅change | 0.70 |
| Mean arterial pressure <40 mmHg  during aortic cross clamp time, min | 1.008 (0.995-1.021) per 1 min increase | 0.21 |
| Mean arterial pressure >90 mmHg during aortic cross‑clamp time, min | 1.035 (1.001-1.068) per 1 min increase | 0.033 |
| SvO^2^ <65% during CPB, min | 1.003 (0.97-1.035) per 1 min increase | 0.87 |
| Flow index during aortic cross-clamp < 2L/min/m2, min | 1.013 (0.988-1.039) per 1 min increase | 0.33 |
| Mean Flow index during CPB, L/min/m^2^ | 2.66 (1.115-6.37) | 0.027 |
| Age |  |  |
| Age <65 years | Reference |  |
| Age 65–74 years | 3.13 (2.1-4.74) | < 0.001 |
| Age ≥75 years | 5.34 (3.43-8.48) | < 0.001 |
| Male sex | 0.88 (0.671-1.161) | 0.36 |
| BMI |  |  |
| BMI <20 kg/m² | 0.775 (0.36-1.53) | 0.49 |
| BMI 20–24.9 kg/m² | 0.75 (0.562-0.996) | 0.048 |
| BMI 25–29.9 kg/m² | Reference |  |
| BMI 30–34.9 kg/m² | 1.339 (0.996-1.794) | 0.05 |
| BMI ≥35 kg/m² | 2.01 (1.258-3.15) | 0.0028 |
| Smoking status |  |  |
| Never smoked | Reference |  |
| Former smoker | 0.929 (0.73-1.184) | 0.55 |
| Current smoker | 0.632 (0.406-0.963) | 0.037 |
| NYHA class |  |  |
| NYHA I | Reference |  |
| NYHA II | 0.656 (0.478-0.903) | 0.0095 |
| NYHA III | 1.185 (0.836-1.682) | 0.34 |
| NYHA IV | 1.737 (0.982-3.02) | 0.05 |
| Diabetes | 1.203 (0.918-1.571) | 0.18 |
| Peripheral vascular disease | 1.427 (0.893-2.23) | 0.13 |
| Hypertension | 1.499 (1.139-1.985) | 0.0043 |
| Glomerular filtration rate |  |  |
| eGFR <30 mL/min/1.73 m2 | 2.13 (1.189-3.76) | 0.0098 |
| eGFR 30–59.9 mL/min/1.73 m2 | 0.647 (0.489-0.851) | 0.0021 |
| eGFR 60–89.9 mL/min/1.73 m^2^ | Reference |  |
| eGFR ≥90 mL/min/1.73 m2 | 2.8 (1.767-4.4) | < 0.001 |
| Critical preoperative event | 2.32 (0.857-6.27) | 0.10 |
| Left ventricular ejection fraction |  |  |
| LVEF Good >50% | Reference |  |
| LVEF Fair 30-49% | 1.869 (1.442-2.41) | < 0.001 |
| LVEF Poor <30% | 2.48 (1.556-3.91) | < 0.001 |
| Operative priority |  |  |
| Elective | Reference |  |
| Urgent | 0.869 (0.657-1.147) | 0.32 |
| Emergency/salvage | 1.262 (0.659-2.33) | 0.47 |
| Cardiac procedure |  |  |
| CABG only | Reference |  |
| Valve only | 0.879 (0.633-1.221) | 0.44 |
| CABG + valve | 0.833 (0.51-1.341) | 0.46 |
| Other/multiple | 1.161 (0.669-1.966) | 0.59 |
| Abbreviations: AKI, acute kidney injury; BMI, body mass index; CABG, coronary artery bypass graft; eGFR, estimated glomerular filtration rate; Hb, haemoglobin; HCT, haematocrit; LVEF, left ventricular ejection fraction; MAP, mean arterial pressure NYHA, New York Heart Association; SvO2, mixed venous oxygen saturation | | |
